# Liver-Targeting Class I Selective Histone Deacetylase Inhibitors Potently Suppress Hepatocellular Tumor Growth as Standalone Agents

**DOI:** 10.3390/cancers12113095

**Published:** 2020-10-23

**Authors:** Subhasish Tapadar, Shaghayegh Fathi, Bocheng Wu, Carrie Q. Sun, Idris Raji, Samuel G. Moore, Rebecca S. Arnold, David A. Gaul, John A. Petros, Adegboyega K. Oyelere

**Affiliations:** 1 School of Chemistry and Biochemistry, Georgia Institute of Technology, 901 Atlantic Drive, Atlanta, GA 30332, USA; stapadar3@mail.gatech.edu (S.T.); sfathi@uchicago.edu (S.F.); bocheng.wu@gatech.edu (B.W.); iraji3@mit.edu (I.R.); smoore83@gatech.edu (S.G.M.); 2 Sophia Bioscience, Inc. 311 Ferst Drive NW, Ste. L1325A, Atlanta, GA 30332, USA; david.gaul@chemistry.gatech.edu; 3 Department of Urology, Emory University School of Medicine, 1365 Clifton Road NE, Atlanta, GA 30322, USA; qcsun@emory.edu (C.Q.S.); rsarnol@emory.edu (R.S.A.); 4 Parker H. Petit Institute for Bioengineering and Bioscience, Georgia Institute of Technology, 315 Ferst Drive, Atlanta, GA 30332, USA

**Keywords:** hepatocellular cancer, histone deacetylase, histone deacetylase isoforms, histone deacetylase inhibitors, zinc binding group, molecular docking, macrolide, orthotopic model, immunoblot, liver tissue accumulation

## Abstract

**Simple Summary:**

Liver cancers are among the leading causes of global cancer deaths. The current therapy options for liver cancers, including hepatocellular carcinoma (HCC), which accounts for over 80% of all cases, have afforded limited benefit to patients with an advanced disease state. HCC results from genetic and epigenetic alterations, including gene-silencing chromatin histone hypoacetylation. The aim of this study was to investigate the potential utility of liver tissue-targeting HDAC inhibitors (HDACi) as a new class of anti-HCC agents. We showed that a class of macrolide-based HDACi, which are selective for sub-class I HDACs, preferentially accumulated in the liver tissue and robustly suppressed tumor growths in an orthotopic model of HCC. The liver tissue-selective accumulation property of these compounds gives them a unique advantage over most of the current HDACi, including those currently in clinical use.

**Abstract:**

Dysfunctions in epigenetic regulation play critical roles in tumor development and progression. Histone deacetylases (HDACs) and histone acetyl transferase (HAT) are functionally opposing epigenetic regulators, which control the expression status of tumor suppressor genes. Upregulation of HDAC activities, which results in silencing of tumor suppressor genes and uncontrolled proliferation, predominates in malignant tumors. Inhibition of the deacetylase activity of HDACs is a clinically validated cancer therapy strategy. However, current HDAC inhibitors (HDACi) have elicited limited therapeutic benefit against solid tumors. Here, we disclosed a class of HDACi that are selective for sub-class I HDACs and preferentially accumulate within the normal liver tissue and orthotopically implanted liver tumors. We observed that these compounds possess exquisite on-target effects evidenced by their induction of dose-dependent histone H4 hyperacetylation without perturbation of tubulin acetylation status and G0/G1 cell cycle arrest. Representative compounds **2** and **3a** are relatively non-toxic to mice and robustly suppressed tumor growths in an orthotopic model of HCC as standalone agents. Collectively, our results suggest that these compounds may have therapeutic advantage against HCC relative to the current systemic HDACi. This prospect merits further comprehensive preclinical investigations.

## 1. Introduction

Liver cancer is among the leading causes of global cancer deaths, with an incidence and mortality two to three times higher among men in most regions of the world [1]. Although the majority of liver cases occur in developing countries, there is an upward trend in liver carcinomas in the developed countries [2,3]. Hepatocellular carcinoma (HCC) is the most common form of liver cancer, accounting for over 80% of all cases, and occurring in >90% of cases in patients with liver damage. The prognosis of HCC is very grim as it has an estimated 18% 5-year survival rate [4]. The treatment option for HCC is surgical resection, which is limited to those patients with good liver function and early stage disease. Unfortunately, only a small proportion of patients are suitable candidates for surgery, and the relapse rate (>60%) is very high after surgery [5]. Liver transplant has proven successful in treating cases of early stage HCC, but only an even smaller proportion of patients are eligible due to the late diagnosis of the disease and limited organ availability [5,6]. Other local strategies, such as transarterial chemo-embolization, radioembolization, and radiofrequency ablation, are largely palliative [7].

Systemic therapy became an option for the treatment of advanced HCC with the FDA approval of sorafenib more than a decade ago, and later of other multikinase angiogenesis inhibitors, such as lenvatinib, regorafenib, and cabozantinib. However, these drugs extend survival in advanced HCC patients only by 1–3 months [8]. Blockade of immune checkpoints (PD-1 alone or with CTLA-4) has also become an option recently, but the vast majority of patients (~70%) still do not respond to immunotherapy, even when combined with antiangiogenic drugs. Therefore, there is a clear need for more efficacious strategies for HCC treatment [9].

HCC is a complex and heterogeneous neoplasm as it results from a large number of genetic and epigenetic alterations. Gene-silencing chromatin histone hypoacetylation plays a significant role in the development and sustenance of HCC [10,11,12,13,14,15,16,17]. Several histone deacetylase (HDAC) isoforms are significantly upregulated in human HCC cell lines and patient tumor samples compared to primary hepatocytes and non-tumorous liver. Specifically, high expression of HDAC1, a member of class I HDAC, has been implicated in the aggressiveness and cell dedifferentiation of HCC [13]. A recent analysis of The Cancer Genome Atlas (TCGA) dataset revealed significant overexpression of HDAC1, HDAC2 (another HDAC class I), and class IIa HDACs-HDAC4, HDAC7, and HDAC9 in high-risk groups. The overexpression of these HDACs strongly correlates with significantly reduced patient survival [14]. A negative correlation between HDAC1 levels and the expression of four microRNAs—miR- 29b, miR- 34a, miR- 449a, and miR- 520h—has been observed in HCC tissues. miR-34a and miR-449a act on the 3′UTR of HDAC1, and their transfection of HCC cells caused downregulation of HDAC1 [15]. Additionally, downregulation of HDACs 1 and 2 exerted anti-proliferative effects in HCC cell lines [16,17]. Hence, small molecules endowed with class I HDAC inhibition activities could offer an attractive therapeutic modality for HCC.

**Figure 1:**
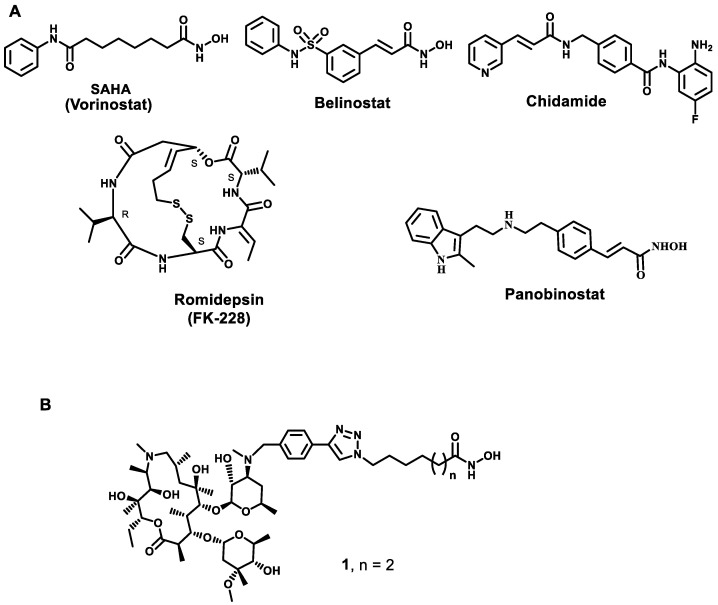
(**A**) Structures of currently approved HDACi (note: optical rotation at Romidepsin chiral centers are indicated with R and S). (**B**) Structure of a representative lead macrolide-based HDACi.

HDAC inhibition is an exciting clinically validated cancer therapy strategy. To date, five HDAC inhibitors (HDACis) (Figure 1) have been clinically approved but all for the treatment of hematologic malignancies [18]. Safer and more effective HDACis are needed to target solid tumors. In HCC, HDACis may suppress HCC progression through multiple mechanisms, including cell differentiation, cell-cycle blockade, induction of alternative pathways of apoptosis, and anti-angiogenic effects [19]. Although HCC cells are sensitive to HDAC inhibition, this has not translated into robust in vivo efficacy using existing agents. As an alternative, HDACis could find use in the clinic against solid tumors as components of drug combination regimens. The combination of HDACi and several standard-of-care agents has resulted in synergistic inhibition of the growth of multiple cancer cell lines. Despite promising preclinical results, several clinical studies of the combination of systemic HDACi with other chemotherapeutic agents in patients with solid tumors have furnished disappointing outcomes [19,20,21]. Bringing to fruition the therapeutic potential of HDACi in HCC and other solid tumors remains of significant interest [22], but this will require improvement of the therapeutic indices of the current HDACi. The poor efficacy of these agents against solid tumors, including HCC, is largely due to their pleiotropic effects, off-target toxicity, and poor biodistribution within solid tumors. These are some of the key challenges whose solution could broaden the clinical application of HDACi to include solid tumors. Here, we disclosed a class of HDACi that are selective for sub-class I HDACs, preferentially accumulate in the liver tissue and are relatively non-toxic to mice. Representative compounds **2** and **3a** induced cancer cell apoptosis via upregulation of p21^WAF1/CIP1^ expression and caspase 3 activation and robustly suppressed tumor growths in an orthotopic model of HCC as standalone agents. Collectively, these HDACi potentially overcome some of the shortcomings that have hindered the utility of the currently available HDACi for the treatment of solid tumors.

## 2. Results

### 2.1. Macrolide-Based Class I HDACi: Design Rationale and Synthesis

Currently, there are 18 known HDAC isoforms, which facilitate the deacetylation of their intercellular targets via Zn^2+^-dependent (class I, II, and IV HDACs) and NAD^+^-dependent (class III HDACs or Sirtuins) mechanisms [23]. The molecular architecture of Zn^2+^-dependent HDACi generally follow a modular three-motif pharmacophoric model consisting of a distinct surface recognition cap group, a linker group, and a zinc binding group (ZBG). When bound to their target HDAC, these HDACi adopt binding orientations, whereby their surface recognition cap group is presented at the enzyme outer rim, the linker group, which connects the surface recognition cap group to ZBG, traverses in the tunnel to optimally position the ZBG for chelation to Zn^2+^ ion at the base of a pocket-like active site [24,25]. Due to its solvent exposure, the surface recognition group is highly tolerant of modifications and has been widely used, in the context of designed multiple ligands [26], to impact new properties to HDACi [27,28,29,30,31,32].

We showed in prior studies that appending macrolides as secondary moieties to the surface recognition cap group of prototypical HDACi resulted in a novel class of non-peptide macrocyclic HDACi [33,34,35]. A secondary characteristic of the macrolide moieties is the propensity of macrolides, such as azithromycin (AZM) and clarithromycin (CLM), to accumulate in resident macrophage-enriched organs, including the lungs, liver, and spleen [36,37,38,39,40]. Thus, AZM and CLM could facilitate the selective delivery of appended chemotypes into these organs [18,41,42]. Specifically, AZM and CLM moieties of the macrolide-based HDACi could facilitate selective drug accumulation within the lung and liver tissues to furnish tissue-targeted HDACi with minimal off-target effects. Such tissue-targeted HDACi could be efficacious against primary and metastized tumors within the liver and lung tissues, and possibly other macrophage-enriched tumors, such as breast tumors in which tumor-associated macrophage constitute up to 50% of the cell mass of the lymphoreticular infiltrates [43].

The macrolide-based HDACi from our previous studies have hydroxamate as a ZBG moiety. Similar to other hydroxamate-based HDACi, these compounds do not to have a strong HDAC isoform selectivity against representative HDACs [33,34,35]. When screened against 11 Zn^2+^-dependent HDACs in Fluor de Lys assay, we confirmed that a representative lead compound **1** elicits pan-HDAC inhibition, inhibiting selected members of class I and II HDACs with single digit nanomolar potency (Table 1). In light of the observed lack of isoform (or subclass) selectivity by these macrolide hydroxamates, we sought to identify sub-class I isoform selective liver-tissue accumulating HDACi. Since class I HDACs are vital to HCC etiology [13], the combined effects of liver tissue accumulation and the inhibition of disease-relevant HDACs could enhance drug efficacy against HCC in vivo.

**Figure 2:**
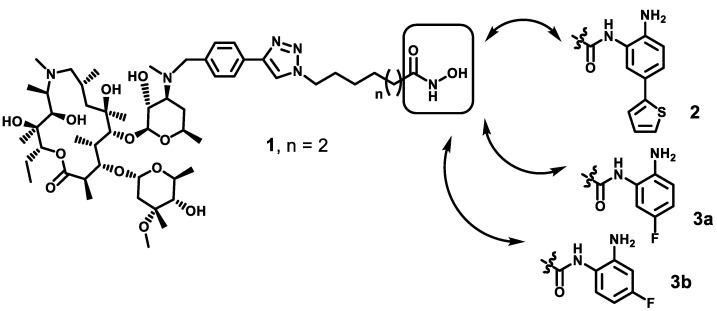
Design of class I selective macrolide-based HDACi **2** and **3a**,**b** based on the swapping of hydrozamate moiety of **1** for class I selective ZBGs.

Most hydroxamate-based HDACi derive a significant part of their binding affinity from the chelation of their ZBG to the active site Zn^2+^ ion. Therefore, swapping of the hydroxamate moiety of optimized pan-HDACi, such as **1**, with class I selective ZBGs, such as the N-(2-amino-phenyl)acylamides (NAPA) [44,45,46,47], should furnish the desired class I selective macrolide-based HDACi **2** and **3a**,**b** (Figure 2). Docking study, using Autodock Vina [48], strongly suggests that the representative NAPA compound **3a** is as well-accommodated as Chidamide, with their ZBGs adopting a nearly identical orientation, at the active site of a selected class I, HDAC2 (PDB: 4LXZ) (Figure S1).

**Scheme 1:**
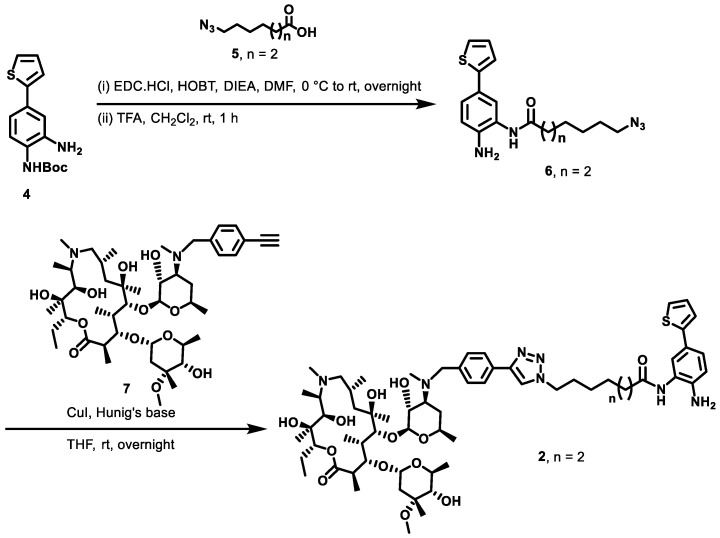
Synthesis of compound **2**.

**Scheme 2:**
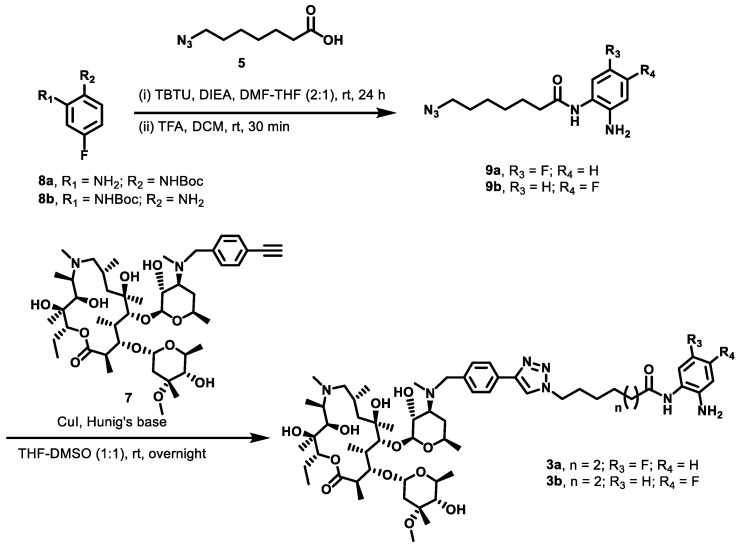
Synthesis of compounds **3a**,**b**.

The synthesis of target compounds **2** and **3a**,**b** was achieved following the reaction routes in Scheme 1 and Scheme 2. EDC-mediated coupling of aniline compound **4** [49] with azido acids **5** [35] followed by Boc-deprotection gave the azido compound **6**. Subsequently, Cu (I)-catalyzed cycloaddition reaction between **6** and alkynyl-AZM **7** [33,34,35] gave the target compound **2** (Scheme 1). Similarly, EDC-mediated coupling of azido acid **5** with aniline compounds **8a**,**b** [50] followed by Boc-deprotection yielded azido compound **9a**,**b**. Cu (I)-catalyzed cycloaddition reaction between **9a**,**b** and alkynyl-AZM **7** gave the target compounds **3a**,**b** (Scheme 2).

### 2.2. In Vitro Profiling, Whole Cell Activity, and Intracellular Target Validation

We first profiled compounds **2** and **3a**,**b** for their HDAC isoform inhibition activities, against class I HDACs 1, 2, 3, and 8; and HDAC6, a representative class IIb HDAC, using fluorescence-based HDAC inhibition assay (BPS Bioscience Inc., San Diego, CA, USA). As anticipated, we observed that these compounds possess varying class I HDAC isoform selectivity (Table 2). Specifically, compound **2** inhibits HDAC1 and HDAC2 with nanomolar and low micromolar IC_50_s, respectively, while devoid of inhibitory activities against class I HDAC8 and class IIb HDAC6. While both compounds **3a**,**b** are inactive against HDACs 6 and 8, they are weaker HDACi and elicit distinct sub-class I selectivity relative to **2**. Both compounds **3a**,**b** inhibit HDAC3, a class I isoform against which **2** is inactive, with low micromolar IC_50_s, and either weakly active (**3a**) or inactive (**3b**) against HDACs 1 and 2. We then selected **2** and **3a** for screening against the remaining HDAC isoforms (HDACs 4, 5, 7, 9, 10, and 11. We observed that, with the exception of HDAC10, which they inhibit with low micromolar IC_50_s, **2** and **3a** are inactive against these HDAC isoforms (Table 2).

Subsequently, we evaluated the effects of pan-HDACi **1** and class I selective compounds **2** and **3a**,**b** on the proliferation of representative HCC cell lines—Hep-G2, Huh-7, SK Hep1, and Hep3B and Kupffer cells—using the WST-1 assay [51]. Our choice of these HCC cell lines was informed by their p53 expression status, which may affect their response to HDAC inhibition. Hep-G2 and Huh-7 express wildtype and mutant p53, respectively, while Hep3B is p53 null. HepG2 is responsive to HDAC inhibition due to its expression of functional p53 [52]. Huh-7 and Hep3B are less responsive to HDACi (not refractory) and are particularly resistant to most cytotoxic drugs because of a lack of functional p53 [53,54,55]. We used Kupffer cells, a non-transformed liver macrophage, to gauge the extent of tumor selectivity of the tested compounds. We observed that all tested compounds have comparable antiproliferative activities against the Hep3B cell line. However, relative to **2** and **3a**,**b**, the pan-HDACi **1** elicits weaker antiproliferative activities against Hep-G2, Huh-7, and SK Hep1 cell lines (Table 3). Among the class I selective compounds, **2** broadly inhibited the proliferation of all the tested HCC cell lines with low micromolar IC_50_s while **3a**,**b** are somewhat less potent. Presumably, the enhanced antiproliferative activity of **2** is a reflection of its more potent sub-class I HDAC inhibition relative to **3a**,**b** (Table 2). Interestingly, these compounds are cancer cell selective as they did not cause a significant reduction in the proliferation of Kupffer cells at the maximum tested concentration of 100 µM.

**Figure 3:**
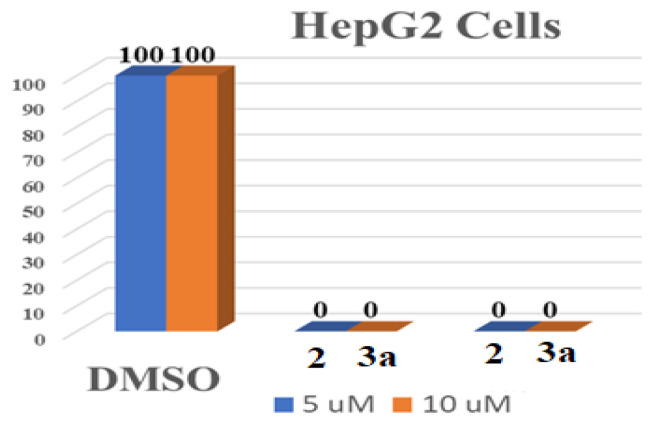
Compounds **2** and **3a** inhibit colony formation by Hep-G2 cells.

To confirm that the metabolic activity determined by the WST-1 assay corresponds with cell viability, we investigated the effects of **2** and **3a** on the viability of HepG2 cells using a standard clonogenic assay (Reaction Biology, Malvern, PA). We observed that **2** and **3a** caused concentration-dependent inhibition of HepG2 cells colony formation (Figure 3).

**Figure 4:**
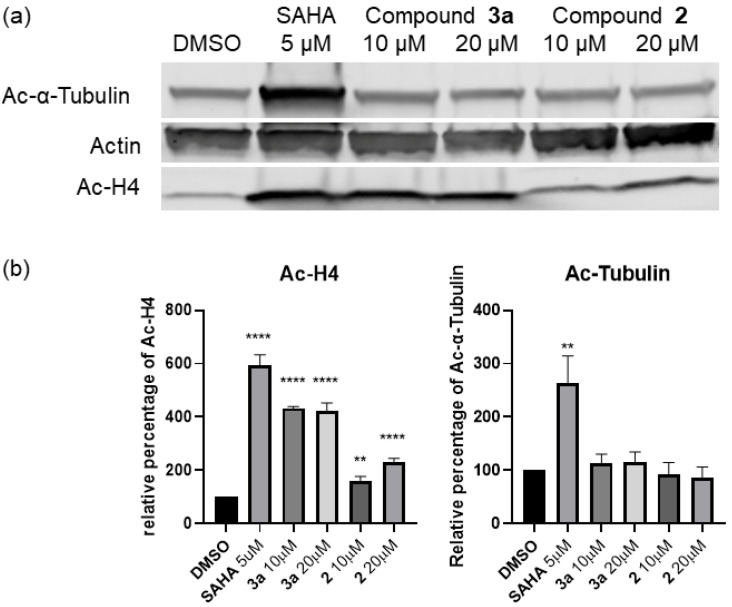
Compounds **2** and **3a** demonstrate selective class I HDAC inhibition in HepG2 cells. (**a**) The immunoblot of acetylated H4 and acetylated α-Τubulin in the Hep-G2 cell line treated with 0.1% DMSO or 0.1% DMSO solution of SAHA (5 μM), **3a** (10, 20 μΜ) and **2** (10, 20 μΜ) for 6 h. (**b**) Quantification of immunoblot obtained, averaging data from two independent experiments. (Bars show mean plus standard deviation; ** *p* < 0.0021; **** *p* < 0.0002).

**Figure 5:**
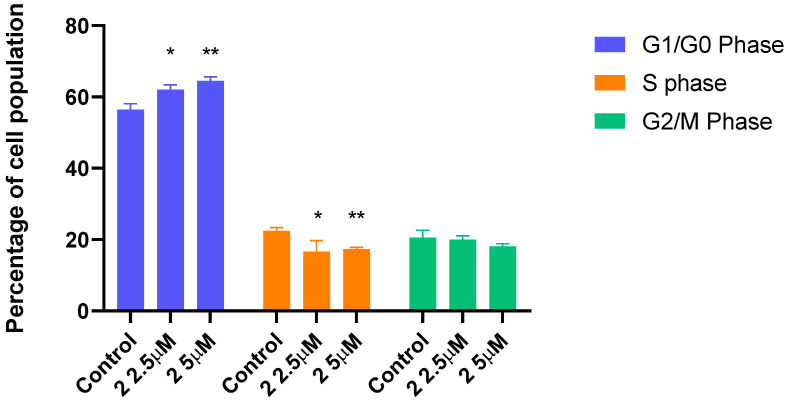
Effect of compound **2** on Hep-G2 cell cycle progression. Analysis of the percentage cell population in cell cycle stages revealed that **2** caused significant G0/G1 arrest. Hep-G2 cells were treated with 0.1% DMSO (control group), compound **2** at 2.5 μM or 5 μM for 48 h prior to cell cycle analysis. Bars show mean plus standard deviation; statistical analysis was performed via two-way ANOVA compared with the control group, * *p* < 0.0332; ** *p* < 0.0021.

**Figure 6:**
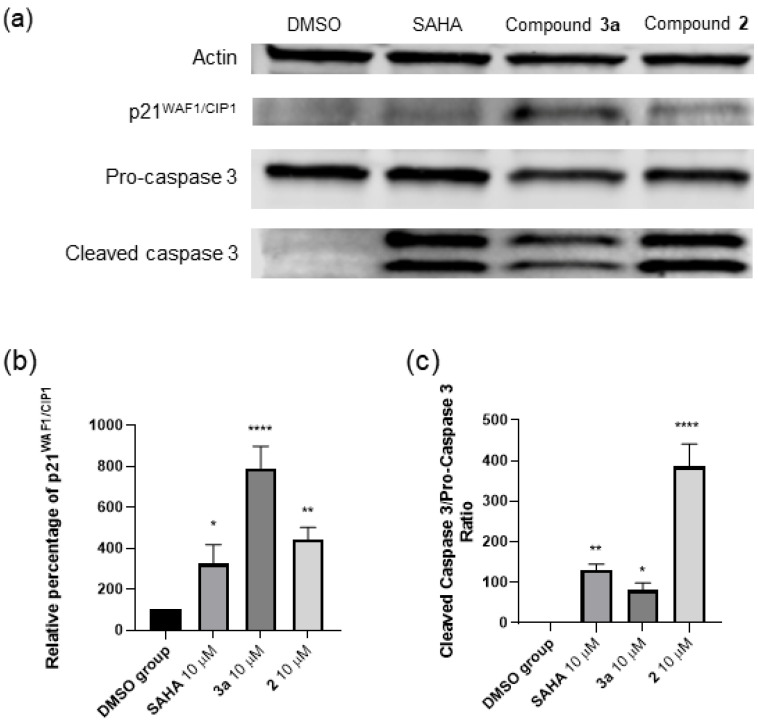
Compounds **2** and **3a** induced apoptosis in the Hep-G2 cell line via p21^WAF1/CIP1^ expression and caspase 3 activation. (**a**) The immunoblot of Actin, p21^WAF1/CIP1^, pro-caspase 3, and cleaved caspase 3 in the Hep-G2 cell line treated with 0.1% DMSO or 0.1% DMSO solution of SAHA (10 μM), **3a** (10 μΜ) and **2** (10 μΜ) for 24 h. Quantification of immunoblot obtained for p21^WAF1/CIP1^ (**b**) and caspase (**c**), averaging data from three independent experiments. (Bars show mean plus standard deviation; * *p* < 0.05; ** *p* < 0.0021; **** *p* < 0.0002).

As part of our study aimed at cellular target and mechanistic validation, we also investigated the effect of a representative compound **2** and **3a** on the acetylation status of histone H4 and tubulin. We observed that **2** and **3a** induced dose-dependent histone H4 hyper-acetylation but no effect on tubulin acetylation status. This data is confirmatory of intracellular HDAC1 inhibition activities of **2** and **3a** (Figure 4). Additionally, cell cycle analysis revealed that **2** caused a significant G0/G1 arrest of Hep-G2 cells (Figure 5 and Figure S2), analogous to the cell cycle arrest caused by other HDACi [56,57]. Moreover, intracellular HDAC inhibition induces apoptosis via re-establishment of the expression of key tumor suppressor proteins, such as p53 and p21^(Cip1/WAF1)^ and caspase 3 activation [18,33]. To investigate if **2** and **3a** possess the cancer cells apoptosis-inducing activities of HDACs, we probed for their effects on the intracellular expression of p21^(Cip1/WAF1)^ and pro-caspase 3 cleavage (caspase-3 activation) via Western blotting. We used SAHA as a control HDACi in these experiments. We observed that in addition to SAHA, **2** and **3a** induced p21^(Cip1/WAF1)^ expression and caspase 3 activation (Figure 6a). While SAHA and **2** induced p21^(Cip1/WAF1)^ to about the same extent at 10 µM, **3a** is twice as effective at this concentration (Figure 6b). Interestingly, compound 2 more effectively induced caspase 3 activation relative to SAHA and **3a** (Figure 6c). Collectively, these data revealed that the anti-proliferative activities of these macrolide-based compounds are largely due to their intracellular HDAC inhibition. Based on these cell data, we focused our in vivo study on the **2** and **3a**.

### 2.3. Maximum Tolerated Dose Determination, In Vivo Efficacy Study, and Tissue Distribution

**Figure 7:**
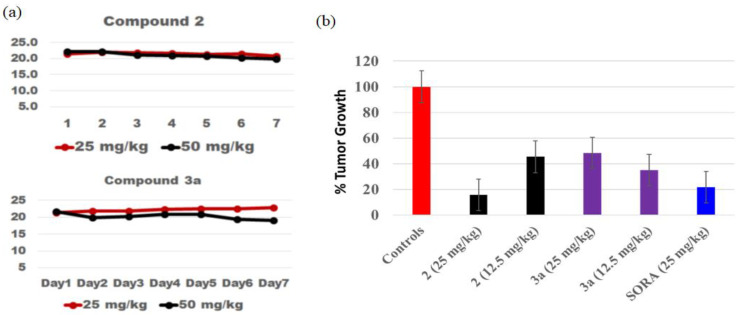
In vivo efficacy of compounds **2** and **3a**. (**a**) Effects of **2** and **3a** on animal weights at the stated doses. (**b**) Compounds **2** and **3a**, as standalone agents, robustly suppressed tumor growth in a murine orthotopic model of Hep-G2-Red-FLuc. Error bars represent 10 mice per cohort with the exception of the control experiments, which had 22 mice in total. Data are representative of two different experiments. *p* values: controls vs. **2** (12.5 mg/kg) = 0.0625 controls vs. **2** (25 mg/kg) = 0.0043; controls vs. **3a** (12.5 mg/kg) = 0.0264; controls vs. **3a** (25 mg/kg) = 0.0888 and controls vs. SORA = 0.0073.

A key concept that we sought to demonstrate in this study is that liver tissue accumulation will enhance the therapeutic indices of HDACi and expand their efficacy in a murine model of HCC. Prior to embarking on tissue distribution and efficacy studies, we first determined the maximum tolerated dose (MTD) in healthy C57Bl/6 mice, for **2** and **3a**. The compounds were administered via i.*p*. injection using a formulation containing excipients found in FDA-approved drugs, dimethylacetamide (DMA)/Cremophor RH 40 (CRH)/Water (10% /20% /70%), that we developed. We exposed cohorts of animals (6 per group, equal number of both sexes) to each drug at three concentrations, 25, 50, and 100 mg/kg (limiting injection volume to 100 µL per dose), daily for 7 days. Using body weight as an indicator of toxicity, we observed no overt toxicity as there was no significant weight loss for **2** and **3a** at 50 mg/kg (Figure 7a). Based on this MTD data, we advanced **2** and **3a** for the in vivo efficacy study.

We challenged compounds **2**, **3a**, and sorafenib (a representative standard of care) to mice bearing an orthotopic model of Hep-G2-Red-FLuc Bioware^®^ Brite Cell Line (PerkinElmer Waltham, MA, USA) (10 per treatment group). The orthotopic model was established by a direct intrahepatic artery injection according to published protocols [58]. We only used the Red-Fluc expression to establish tumor implantation and not to infer treatment efficacy due to the upregulation of luciferase expression previously noted with HDACi treatment in an HCC model [53]. Specifically, one week after cells were injected into the liver, the level of chemiluminescence was measured using IVIS. The average relative value of luminescence per mouse was 3.51E + 6 and the mice were stratified into cohorts with similar average relative chemiluminescence. Treatment began after bioluminescent imaging confirmed the establishment of intrahepatic tumors (approximately 7 days). In the first set of experiments, the treatment groups were administered test compounds **2** and **3a** (12.5 and 25 mg/kg), and sorafenib (25 mg/kg) via the i.*p*. route. Our choice of the tested dosages was informed by the results from the MTD study (Figure 7a) and literature data on sorafenib [59]. Animals were dosed once a day for 21 days and imaged again prior to sacrifice at the end of day 21. We harvested the livers and determined the post-treatment tumor size by measurement with calipers. We calculated the tumor volume using a standard formula: width^2^ × length × 0.52 [53], and compared the mean final tumor volumes of the treatment group with the control animals to determine the statistical significance of treatment. We observed that sorafenib caused tumor growth inhibition (TGI) of 78% (Figure 7b and Figures S3 and S4). This observation is confirmatory of the literature data on sorafenib, which has been shown to inhibit the growth of tumors derived from HepG2 cells in murine models [55,59]. Gratifyingly, compounds **2** and **3a** displayed dose-dependent tumor growth inhibition, inducing TGIs of 53% and 66%, respectively, at 12.5 mg/kg. At 25 mg/kg, **2** and **3a** induced TGI of 83% and 55%, respectively, with **2** somewhat more potent than sorafenib (Figure 7b).

**Figure 8:**
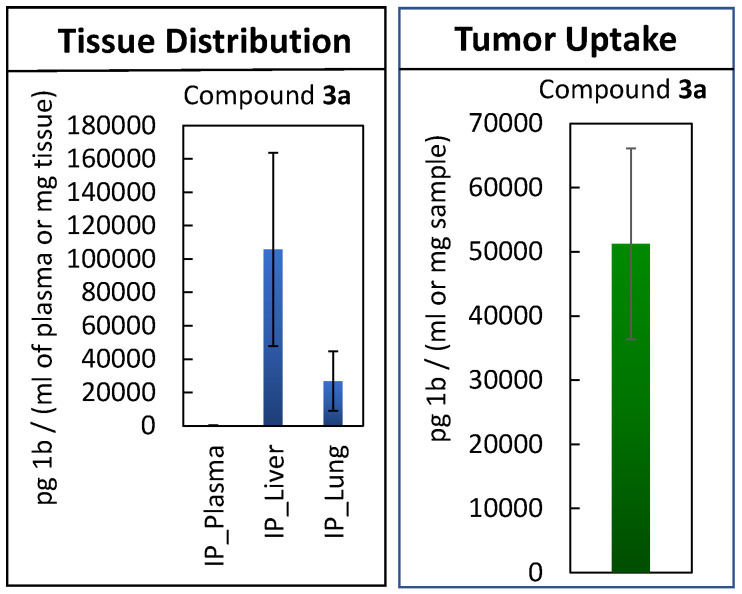
Analysis of tissue distribution and tumor uptake of **3a**. Proteins were precipitated from serum by treatment with LC-MS grade methanol in a 1:3 ratio. Liver, lung, and tumor samples were mixed with 80% methanol (10 mg:400 µL), homogenized, and the insoluble materials were pelleted by centrifugation to separate the supernatants. All samples were stored at 4 °C until analysis. The levels of **3a** in each sample were determined using ultra-performance liquid chromatography coupled to mass spectrometry (UPLC-MS).

To determine if liver tissue accumulation plays a role in the observed in vivo efficacy of these macrolide-derived HDACi, we used mass spectrometric tools to measure the levels of representative compound, **3a**, in selected tissues: liver, lungs, and the plasma. We first analyzed samples from healthy mice exposed to **3a** (25 mg/kg, IP) for 8 h and representative mice from post-efficacy study (25 mg/kg). In the plasma, the level of **3a** is below the detection limit of our instrument (902 pg/mL). The liver/plasma, tumor/plasma, and lung/plasma ratios of **3a** are >110, >55, and >33, respectively (Figure 8). More importantly, the liver levels of **3a** are 9–20-fold higher than the IC_50_ of inhibition of all the HCC cells that we tested.

## 3. Discussions

HCC is driven by genetic mutations and epigenetic dysfunctions, including gene-silencing chromatin histone hypoacetylation [10,11,12,13,14,15,16,17]. The vital roles of HDACs, particularly sub-members of class I HDAC, in the development and sustenance of HCC makes HDAC inhibition a potentially valid therapeutic strategy for HCC. However, despite their success in hematologic cancer, the therapeutic application of HDACi against HCC and other solid tumors has not been realized. To fully harness their therapeutic potential against solid tumors, there is a need to significantly change the current paradigm of HDACi design approaches.

We showed previously that macrolides AZM, CLM, and their analogs are excellent surrogates for the depsipeptide moieties of macrocyclic HDACi, furnishing a new class of pan-HDACi [33,34,35]. Herein, we used our observations from these prior studies to design HDAC sub-class I isoform selective macrolide-based HDACi. Because class I HDACs 1 and 2 are vital to HCC viability, these class I HDAC selective macrolide-based agents could be better suited for HCC than pan-HDACi. Another characteristic of the AZM and CLM is their ability to selectively accumulate in tissue, such as the liver, lungs, and spleen. If this selective tissue distribution property is preserved in the macrolide-based HDACi, they could constitute a new class of HDACi with intrinsic ability to selectively accumulate at the sites of hard-to-treat solid tumors, such as HCC and lung cancer.

We observed that relative to the pan-HDACi **1**, the class I selective HDACi **2** and **3a** more effectively inhibited the proliferation of HCC cell lines in the WST-1 assay regardless of their p53 expression status. The inhibition of HepG2 colony formation (Figure 3) further confirmed that the metabolic activity determined by the WST-1 assay corresponds with the inhibition of cell viability by **2** and **3a**. Moreover, **2** and **3a** demonstrated a strong on-target effect as evidenced by their induction of dose-dependent histone H4 hyperacetylation without perturbation of the tubulin acetylation status and G0/G1 cell cycle arrest. Compounds **2** and **3a** also induced apoptosis in the Hep-G2 cell line via upregulation of p21^WAF1/CIP1^ expression and caspase 3 cleavage (Figure 6). In addition to supporting the importance of class I HDACs in HCC etiology, these data revealed that the anti-proliferative activities of **2** and **3a** are largely due to their intracellular HDAC inhibition. In vivo efficacy study, using murine HCC orthotopic model, revealed that the on-target effects of **2** and **3a** translated into a robust tumor growth suppression that is comparable or better than the effect of sorafenib, the first multikinase angiogenesis inhibitor approved for HCC (Figure 7). Subsequent analysis of selected tissues from healthy and tumor-bearing mice revealed that the tissue-selective accumulation of the parent AZM template was preserved as the representative compound **3a** selectively accumulated within the liver, lungs, and tumors relative to the plasma (Figure 8). The enhanced liver tissue and/or intratumoral uptake most likely contributed to the improved efficacy of these macrolide-based HDACi relative to prototypical HDACi SAHA, which did not fare as well in a similar in vivo model [53].

Earlier literature observations have provided hints about the potential advantage of liver tissue accumulation of HDACi in the enhancement of their tumor growth inhibition and/or suppression of metastatic spread of liver tumors. Specifically, intra-tumoral administration of 4-phenylbutyrate (a short-chain fatty acid HDACi), via an intra-tumor catheter, caused a reduction in the growth of xenograft tumors derived from HCC cell lines [60]. Additionally, polymer-based HDACi pro-drug (Hyaluronate-Butyrate), designed to target membrane receptor CD44 on liver sinusoidal epithelial cells, provided further indication that HDACi liver accumulation may represent a promising approach for HCC treatment as inhibition of tumor growth and metastatic spread was observed [61]. The compounds disclosed herein are neither pro-drugs that must fall apart intracellularly to present antitumor activity, nor do they need to be administered through intrusive delivery devices. Rather, they are HDACi endowed with liver tissue-selective accumulation property, a unique advantage over most of the current HDACi, including those currently in clinical use.

## 4. Materials and Methods

### 4.1. Chemical and Reagents

Anhydrous solvents and reagents were purchased from Sigma-Aldrich (St. Louis, MO, USA), Acros, VWR International (Radnor, PA, USA), Greenfield Chemicals, or Thermo Fisher Scientific (Waltham, MA, USA) and were used without further purification. Analtech silica gel plates (60 F254, Millipore Sigma, Burlington, MA, USA) were utilized for analytical TLC, and Analtech preparative TLC plates (UV254, 2000 μm, Millipore Sigma) were used for purification. Silica gel (200−400 mesh) was used in column chromatography. TLC plates were visualized using UV light, anisaldehyde, and/or iodine stains. High-performance liquid chromatography (HPLC) analyses were performed on an Agilent 1260 Infinity II instrument using a Luna^®^ 5 µm C-18 column 100 Å (100 mm × 4.6 mm, Phenomenex, Torrance, CA, USA) eluted with solvent A (water) and solvent B (acetonitrile) at a gradient of 5% solvent B for the first 4 min and increased to 40% from 4 to 5 min, then eluted with a gradient from 40% to 80% for another 20 min, and then constantly eluted with 80% solvent B for 5 min. The detection wavelength was 280 nm and a flow rate of 0.5 mL/min. Sample concentrations were 250 μM- 1 mM, injecting 30 μL. All compounds had ≥95% purity as determined by HPLC. Chemicals used in the LC-MS analyses were obtained from the following sources: acetonitrile (Optima, LC-MS, Thermo Fisher Scientific, Catalog No. A955–4); formic acid (Optima, LC-MS, Thermo Fisher Scientific, Catalog No. A117–50); isopropanol (Optima, LC-MS, Thermo Fisher Scientific, Catalog No. A461–4); water (Optima, LC-MS, Thermo Fisher Scientific, Catalog No. W6–4); and methanol (Optima, LC-MS, Thermo Fisher Scientific, Catalog No. A456–4). NMR spectra were obtained on a Varian-Gemini 400 MHz magnetic resonance spectrometer. ^1^ H NMR spectra were recorded in parts per million (ppm) relative to the residual peaks of CHCl_3_ (7.24 ppm) in CDCl_3_ or CHD_2_OD (4.78 ppm) in CD_3_OD or DMSO-*d5* (2.49 ppm) in DMSO-*d6*. ^13^C spectra were recorded relative to the central peak of the CDCl_3_ triplet (81.5 ppm) and were recorded with complete hetero decoupling. MestReNova (version 11.0, Mestrelab Research Compostela, Spain) was used to process the original NMR “fid” files. High-resolution mass spectra were recorded at the Georgia Institute of Technology mass spectrometry facility.

Cell culture reagents were from ATCC (Manassas, VA, USA), Sekisui XenoTech, LLC (Kansas City, KS, USA) and Corning Life Sciences (Tewksbury, VA, USA). Cell lines were purchased from ATCC (Hep-G2 and SK Hep1) and Sekisui XenoTech, LLC (Huh-7 and Kupffer cells). All cells were cultured in media recommended by the suppliers: Hep-G2 and SK Hep1in ATCC-formulated EMEM (ATCC^®^ 30–2003™); Huh-7 in DMEM (containing 4 mM L-glutamine and 1 g/L glucose) with 10% FBS (GIBCO Cat. #10099, Thermo Fisher Scientific); and Kupffer cells in OptiThaw Kupffer cell thaw/culture media (Sekisui XenoTech, LLC, Cat. #K7800). Electrophoresis supplies, TGX MIDI 4–20% gel (cat. #5671093) and Turbo PDVF membrane (cat. # 1704273), were from Bio-Rad Laboratories, Inc (Hercules, CA, USA). Primary antibodies, Ac-Tubulin (sc-23950), Ac-H4 (sc-515319), were obtained from Santa Cruz Biotechnology (Dallas, TX, USA) while secondary antibody (part. IR2173) was from ImmunoReagents (Raleigh, NC, USA). RIPA buffer (VWRVN653-100ML) was from VWR International (Radnor, PA, USA) while phosphatase inhibitor (A32957) and protease inhibitor (A32955) were procured from Thermo Fisher Scientific. The BCA protein assay kit (K813-2500) was purchased from BioVision, Inc (Milpitas, CA, USA). C57BL/6 and Athymic Nude-Foxn1nu mice (5–7 weeks old; male and female) were purchased from Envigo RMS, Inc (Indianapolis, IN, USA).

### 4.2. Molecular Docking and Compound Synthesis

A docking study was performed, using Autodock Vina [48], on the crystal structure of HDAC 2 (4LXZ) (Figure S1) as we described before [29,56]. Details about compound synthesis and characterization are provided in the Supplementary Materials.

### 4.3. HDAC Assay

The HDAC inhibition assay was performed at BPS Bioscience, San Diego, CA. Detailed protocol for this experiment is included in the Supplementary Materials.

### 4.4. Cell Culture and Viability Assay

Cells were plated into 96-well plates and treated with DMSO, or 1 to 100 µM drug when the cells reached 70 % confluency for 72 h prior to the cell viability assay. Cell viability was measured using Cell Proliferation Reagent WST-1 (Sigma-Aldrich). Briefly, after the addition of 10 µL reagent per well, plates were incubated for 45 min at 37 °C. Colormetric analysis was performed by measuring the signal at 450 and 690 nm. Data was analyzed according to the manufacturer’s protocol. Six data points were averaged per concentration per cell line.

### 4.5. Cell Colony Formation and Hep3B Cell Viability Assay

The effects of compounds on HepG2 cells’ colony formation and viability of Hep3B cells were performed through contractual agreement with Reaction Biology Corp (Woodbridge, CT, USA). Detailed protocols for these experiments are included in the Supplementary Materials.

### 4.6. Western Blot Analysis

Hep-G2 cells were seeded into a 6-well plate at 1 × 10^6^/well in EMEM for 24 h. Then, 5 μM SAHA (positive control) and **2** or **3a** with 10 or 20 μΜ solutions in DMSO were added to the cell culture media to give a final DMSO level of 0.1%. Cells were treated for 6 h, washed with cold PBS, and lysed with 110 μL RIPA buffer containing phosphatase and protease inhibitors. The cells were scraped, and the lysates were collected and vortexed for 15 s followed by sonication for 60 s. The lysates were centrifuged (14,000 rpm for 15 min) and the supernatants were collected. Subsequently, the total protein concentrations in the supernatants were determined using a BCA protein assay kit. The lysates were diluted to make an equal protein concentration and 20–40 μg of each lysate were loaded per well of a TGX MIDI 4–20% gel and electrophoresis was ran at 150 V for 70 min. The gel was electro-blotted on to the Turbo PDVF membrane (Bio-Rad, 1704273). After blocking with 5% BSA for 1–2 h, the PDVF membrane was incubated with Ac-Tubulin, Ac-H4, Bcl-2, and Bcl-xL antibodies. After incubation overnight, the membrane was washed with TBST (3 × 5 min). Secondary antibody was added, and the membrane was incubated with agitation for 1 h. Bands were quantified using the Odyssey CLx Image system.

### 4.7. Flow Cytometry

Hep-G2 cells were cultured until 80% confluence in a 10-cm petri dish. The cells were serum starved overnight before drug treatment. Then, the cells were treated with 10 mL of 0.1% DMSO medium (control), 0.1% DMSO solution of 5 µM SAHA, or 2.5 µM or 5 µM **2** for 48 h. Cells were trypsinized, washed with cold 1 × PBS solution twice, collected using 1 × PBS buffer, and fixed overnight at −20 °C using 70% ethanol. Subsequently, cells were washed again, centrifuged, and re-suspended in 1 × PBS. The suspension was treated with 200 µg/mL RNaseA for 30 min, followed by treatment with 50 µg/mL PI staining solution at room temperature for 30 min. Cell cycle was analyzed with a BD FACS Aria-Illu Analyzer and the data processed using FlowJo.

### 4.8. Maximum Tolerated Dose Determination

All mouse experiments were done with full IACUC approval. C57BL/6 mice were purchased from Envigo. Mice were 6–8 weeks old, 3 males and 3 females per treatment group. Compounds at concentrations of 20, 50, and 100 mg/kg were injected intraperitoneally once daily for 7 days in Kolliphor/DMA/water vehicle. Body weight, general disposition, food consumption, and mortality were monitored daily. All mice were sacrificed by cervical dislocation on day 8, 24 h post the last dosage. Organs were harvested and frozen at −80 °C.

### 4.9. Tissue Distribution Studies

C57BL/6 mice were purchased from Envigo. Mice were 6–8 weeks old, 3 males and 3 females per treatment group. Compounds were injected into mice once at a concentration of 50 mg/kg iv and/or ip in Kolliphor/DMA/water vehicle. Organs were harvested and frozen at −80 °C 8 h post injection for subsequent quantification by LC-MS analyses.

### 4.10. Liquid Chromatography-Mass Spectrometry

Mouse serum was mixed with LC-MS grade methanol in a 1:3 ratio to precipitate proteins. The excised liver, lung, or tumor were weighed and extracted with a constant ratio of 80% methanol (10 mg:400 µL) and homogenized with 3-mm tungsten carbide beads and a Qiagen Tissuelyser II. The insoluble material was pelleted with centrifugation and the supernatant was stored at 4 °C until analysis. Five dilutions of the standard compound were prepared in 80% methanol. Samples were analyzed once while the standards were injected in triplicate.

Ultra-performance liquid chromatography coupled to mass spectrometry (UPLC-MS) was performed using a Vanquish (Thermo Fisher Scientific), fitted with a Waters Corporation CORTECS T3 column (2.1 × 150 mm, 1.6 µm particle size), coupled to a high-resolution accurate mass Q Exactive HF Hybrid Quadrupole-Orbitrap mass spectrometer system (Thermo Fisher Scientific). The chromatographic method for sample analysis involved elution with H_2_O and 0.1% formic acid (mobile phase A) and 50:50 acetonitrile:isopropyl alcohol, with 0.1% formic acid (mobile phase B) using the following gradient program: held at 80% A from 0 to 0.2 min; 3 min 60% A; 4 min 0% A; held at 0% until 7.0 min; 7.2 min 80% A; and held until 9 min. The flow rate was 0.30 mL min^−1^ for 0 to 4.5 min; increased to 0.4 mL min^−1^ by 4.8 min; held constant until 8.5 min; and decreased to 0.3 mL min^−1^ by 9 min. The column temperature was set to 50 °C, and the injection volume was 2 µL.

The Q Exactive HF Hybrid Quadrupole-Orbitrap mass spectrometer (Thermo Fisher Scientific) utilizes quadrupole isolation and an orbitrap detector, with a maximum resolving power of 240,000 FWHM at m/z 200 and a mass accuracy of <3 ppm. The heated electrospray ionization (HESI) source was operated at a vaporizer temperature of 275 °C, a spray voltage of 2.8 kV, and sheath, auxiliary, and sweep gas flows of 60, 18, and 4, respectively. The 1128.7 564.9 m/*z* transition was measured from 1.5 to 3.5 min with a 30,000 resolution, 0.4 m/*z* isolation window, and 20 normalized collision energy. Xcalibur was used to integrate the peak areas. The limit of detection (LOD) for plasma and tissue samples were 902 pg/mL and 36 pg/mg of tissue, respectively.

### 4.11. In Vivo Efficacy Studies

Athymic nude mice were purchased from Envigo. Mice were 6–8 weeks old, with a body weight of 17 g or above. The orthotopic mouse model of HCC was induced using the protocol described by Reilberger et al. [58]. Briefly, after anesthetizing the animal, the liver was partially exposed, and cells suspended in 20 uL of a serum-free medium containing 50% of Matrigel were injected in the subcapsular region on the left hepatic lobe. Each animal received one million cells. The incision was closed, and the animal allowed to recover. Tumor growth was evaluated with the IVIS Spectrum Series pre-clinical optical Imaging System (PerkinElmer). Briefly, 100 mg/kg D-luciferin per animal was injected interperitoneally 7 min prior to imaging. The first imaging was performed on day 7 after surgery. The last imaging was taken at 24 h after the last drug dosage administration. Beginning at day 8 after tumor cell implantation, male and female mice were treated with individual compound or sorafenib. Kolliphor/DMA/water vehicle was used as a reagent control. Mice were treated daily by intraperitoneal injection of 200 µL of compound solution for a total of 21 days. Tumors were measured at 24 h after the last dosage administration.

## 5. Conclusions

In conclusion, we disclosed herein a class of HDACi that are selective for sub-class I HDACs and preferentially accumulate within the liver tissue and orthotopically implanted liver tumor. Our results suggest that these compounds may be more efficacious against HCC than current systemic HDACi [19,20,21], either as standalone agents or possibly in combination with standards of care, such multikinase angiogenesis inhibitors. These compounds have the potential to positively impact HCC therapy. This prospect merits further comprehensive preclinical investigations.

## Figures and Tables

**Table 1:** Inhibition activities of lead compound **1** against 11 Zn^2+^-dependent HDAC isoforms.

Enzymes	1 (IC_50_, nM)
HDAC1	2.7
HDAC2	8.1
HDAC3/NCOR2	1.7
HDAC4	1904
HDAC5	1773
HDAC6	1.7
HDAC7	2621
HDAC8	839
HDAC9	6910
HDAC10	4.5
HDAC11	7361

**Table 2:** HDAC isoform inhibition activities of **2** and **3a**,**b**.

Enzymes	2 (IC_50_, nM)	3a (IC_50_, nM)	3b (IC_50_, nM)
HDAC1	509	3800	NI
HDAC2	3454	9200	NI
HDAC3/NCOR2	NI	3000	2400
HDAC4	NI	NI	NT
HDAC5	NI	NI	NT
HDAC6	NI	NI	NI
HDAC7	NI	NI	NT
HDAC8	NI	NI	NI
HDAC9	NI	NI	NT
HDAC10	2285	3000	NT
HDAC11	NI	NI	NT

NI = No inhibition at maximum tested concentration; NT = Not Tested.

**Table 3:** Antiproliferative effects of pan-HDACi **1** and class I selective compounds **2** and **3a-b** against HCC cell lines.

	Cell Lines IC_50_ (µM)				
Compounds	Hep-G2	Huh7	SK-HEP1	Hep3B	Kupffer
**1**	16.08	25.54	28.21	2.82	>100
**2**	3.27	1.48	3.08	3.51	>100
**3a**	6.76	3.82	3.83	4.61	>100
**3b**	6.93	3.56	9.02	6.60	NT

NT = Not tested.

## Supplementary Materials

The following are available online at https://www.mdpi.com/2072-6694/12/11/3095/s1. Figure S1: Molecular docking analysis, using Autodock Vina; Figure S2: Flow cytometry data showing the effects of compound 2 on Hep-G2 cell cycle progression.; Figure S3: Bioluminescent imaging of mice before the commencement of experiments; Figure S4: Post-experiment bioluminescent imaging mice (images were captured at day 22nd just before tumors were excised) and pictures of excised mice livers.; Compound Synthesis.
