# Supplementary material for: Liver-Targeting Class I Selective Histone Deacetylase Inhibitors Potently Suppress Hepatocellular Tumor Growth as Standalone Agents
Source: Cancers (Basel). 2020 Oct 23;12(11):3095. doi: 10.3390/cancers12113095 (PMC7690782; doi:10.3390/cancers12113095)
Supplement: Supplementary file 1 [file cancers-12-03095-s001.pdf]

Liver-Targeting Class I Selective Histone Deacetylase Inhibitors Potently Suppress Hepatocellular Tumor Growth as Standalone Agents.

Subhasish Tapadar[1, 2], Shaghayegh Fathi[1±¶], Bocheng Wu[1¶], Carrie Q. Sun[3], Idris Raji [1†], Samuel G. Moore[1], Rebecca S. Arnold[3], David A. Gaul[2], John A. Petros[3*] and Adegboyega K. Oyelere[1, 2, 4*]

1. School of Chemistry and Biochemistry, Georgia Institute of Technology, Atlanta, GA 30332-0400, USA; stapadar3@mail.gatech.edu (S.T.); sfathi@uchicago.edu (S.F.); bocheng.wu@gatech.edu (B.W.); iraji3@mit.edu (I.R.); smoore83@gatech.edu (S.G.M.); david.gaul@chemistry.gatech.edu (D.A.G.)

2. Sophia Bioscience, Inc. 311 Ferst Drive NW, Ste. L1325A, Atlanta, GA 30332, USA.

3. Department of Urology, Emory University School of Medicine, Atlanta, GA 30322, USA; qcsun@emory.edu (C.Q.S.); rsarnol@emory.edu (R.S.A.)

4. Parker H. Petit Institute for Bioengineering and Bioscience, Georgia Institute of Technology, Atlanta, GA 30332-0400, USA.

[*]Correspondence: jpetros@emory.edu (J.A.P.); aoyelere@gatech.edu (A.K.O.); Tel: +404-894-4047 (A.K.O.)

[¶]These authors contributed equally to the manuscript.

[±]Present Address: Department of Chemistry, University of Chicago, Chicago, IL 60637, USA.

[†]Present Address: Koch Institute for Integrative Cancer Research, Massachusetts Institute of Technology, Cambridge, MA 02139, USA.

Supporting Information

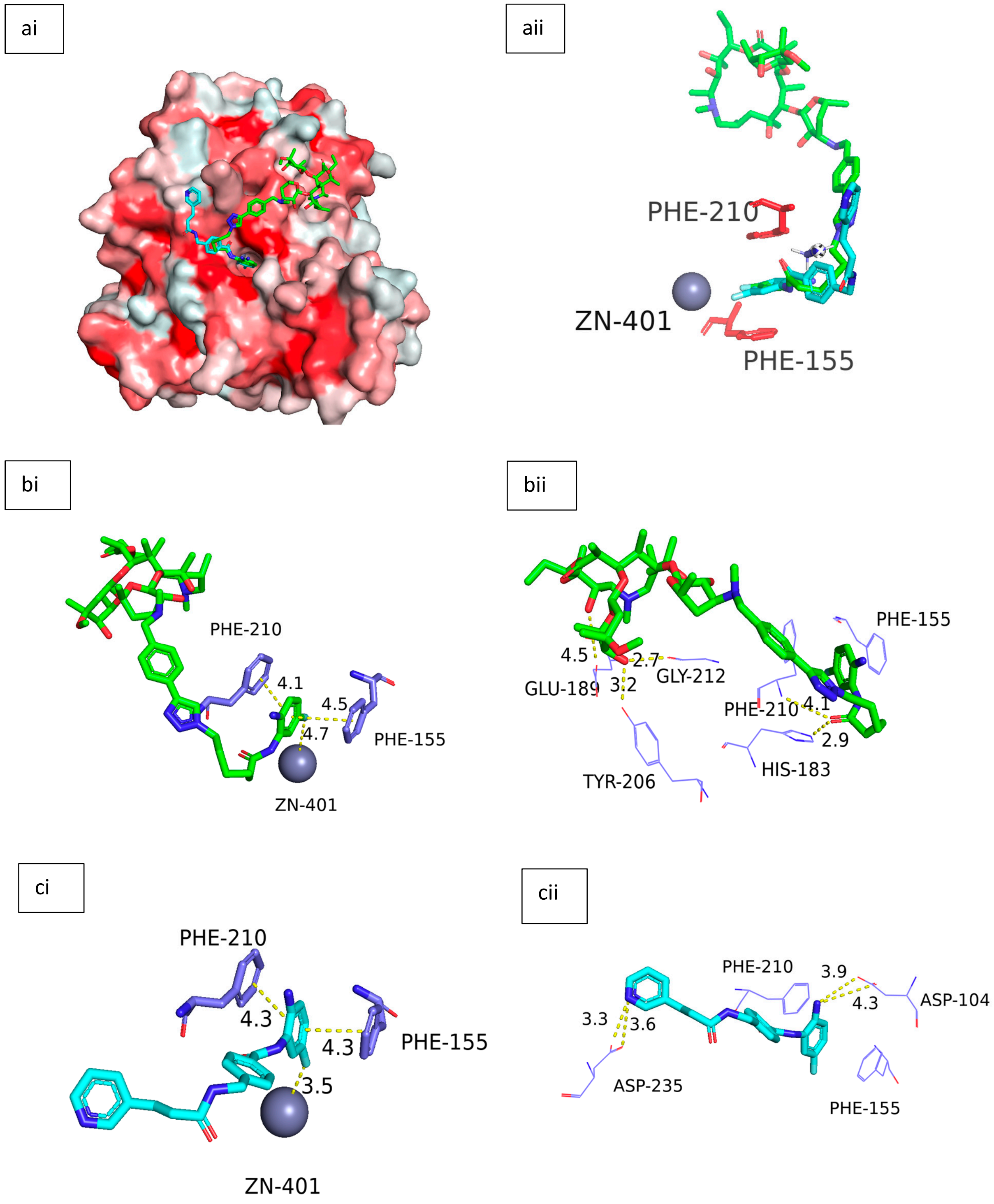

Figure S1: Molecular docking analysis, using Autodock Vina [48], revealed that compound **3a** and Chidamide productively interact with (PDB: 4LXZ). (ai) Overlay of the docking outputs of **3a** (green) and Chidamide (Cyan) at the active site HDAC2 shown as surface presentation; (aii) Illustration of orientation of **3a** and Chidamide focusing on the HDAC2 active site Zn^{2+}-ion and key common interacting residues. (bi) Compound **3a** HDAC inhibition moiety binds to the HDAC2 active site through stacking interaction between Phe-155 and Phe-210, and chelation to Zn^{2+}-ion; (bii) Evidence for additional interaction of the macrolide moiety of **3a** with HDAC2 outer-rim through possible H-bonding with His-183, Glu-189, Tyr-206 and Gly-212. (ci) In analogous manner as **3a**, Chidamide is accommodated at HDAC2 active site through stacking interaction between Phe-155 and Phe-210, and chelation to Zn^{2+}-ion; (cii) Evidence showing that Chidamide could form H-bonding interactions with Asp-235 and Asp-104.

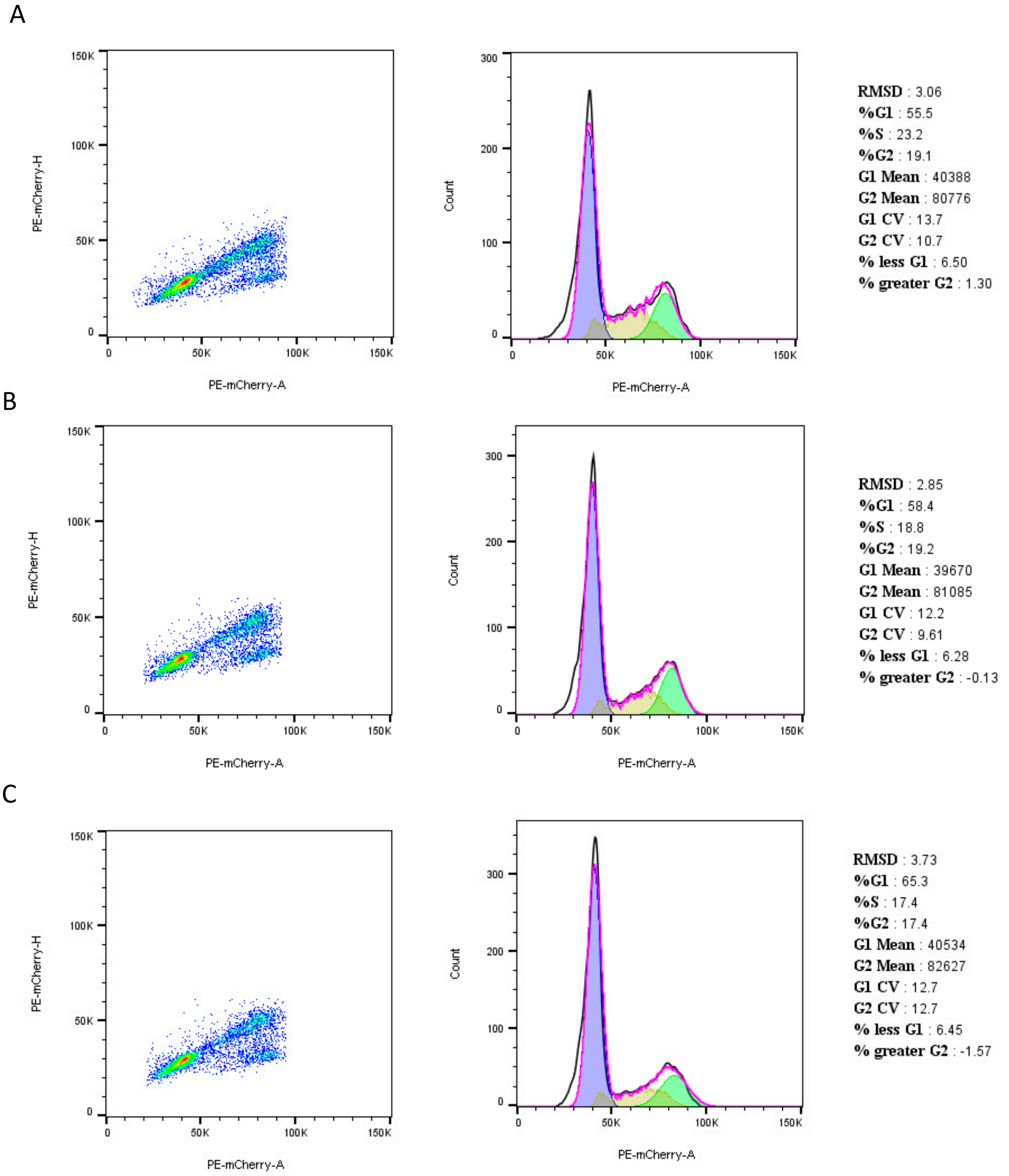

Figure S2: Flow cytometry data showing the effects of compound **2** on Hep-G2 cell cycle progression. (a) Control group with 0.1% DMSO, (b) **2** at 2.5µM and (c) **2** at 5µM.

Male (Control)

B0 B3 D0 D3 D30

Female (Control)

F3 F30 F33 H30 H33

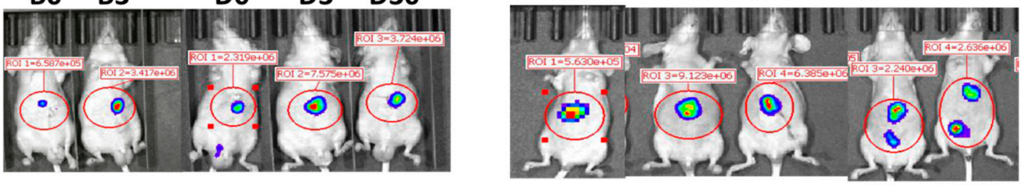

25% Kolliphor RH40 and 10% DMA

Male

0 3 30 33 5

Female

0 3 30 33 5

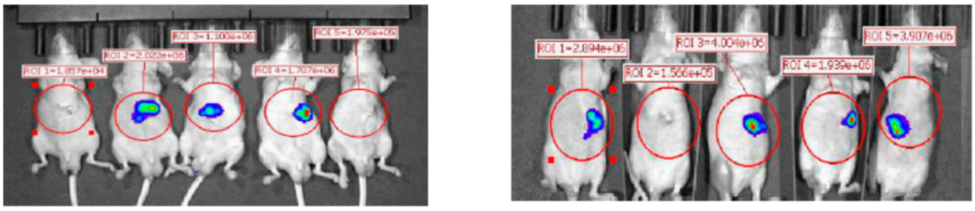

Sorafenib (dosage: 25 mg/kg)

Male

0 3 30 33 5

Female

0 3 30 33 5

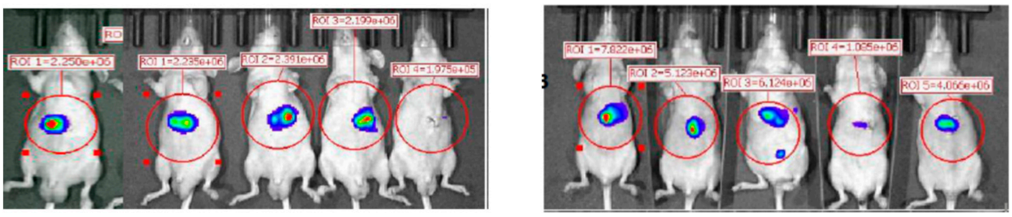

Compound 2 (dosage: 12.5 mg/kg)

H0 H3 H30 H33 E3

B0 B30 B33 B5 C30

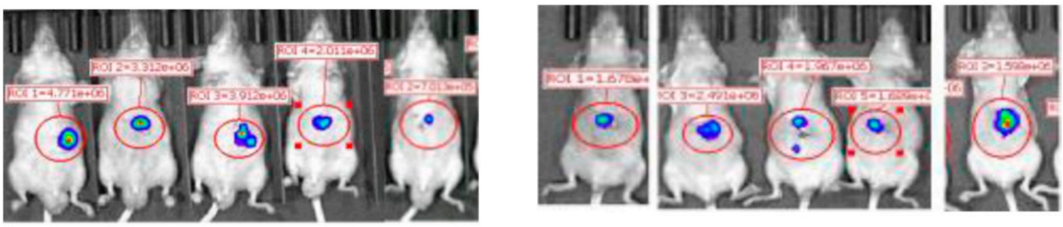

Compound 2 (dosage: 25 mg/kg)

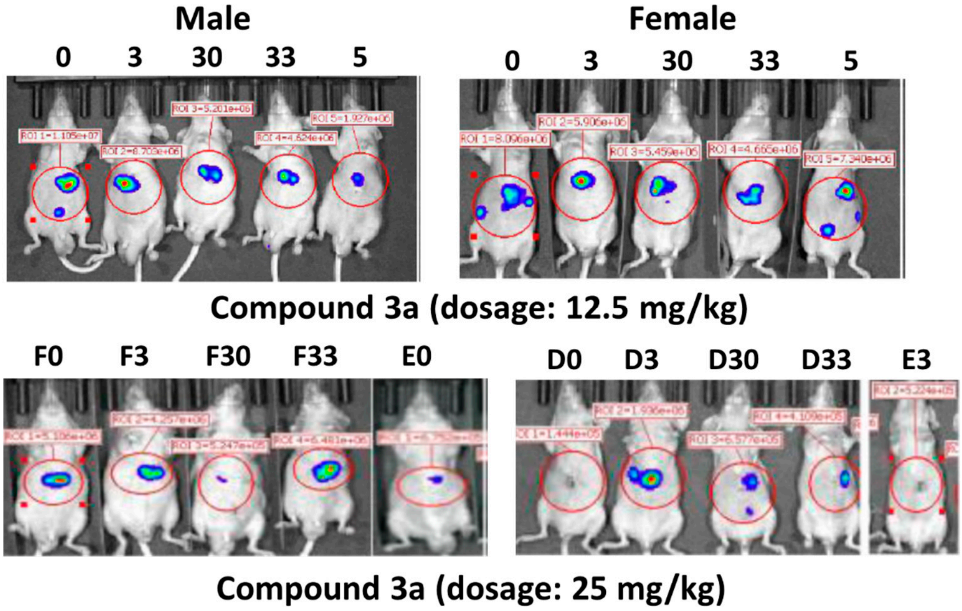

Figure S3: Bioluminescent imaging of mice before the commencement of experiments to verify tumor implantation of control, sorafenib, **2** and **3a** (images were captured at day 7th after injecting the cell into the mice and before the start of the treatment).

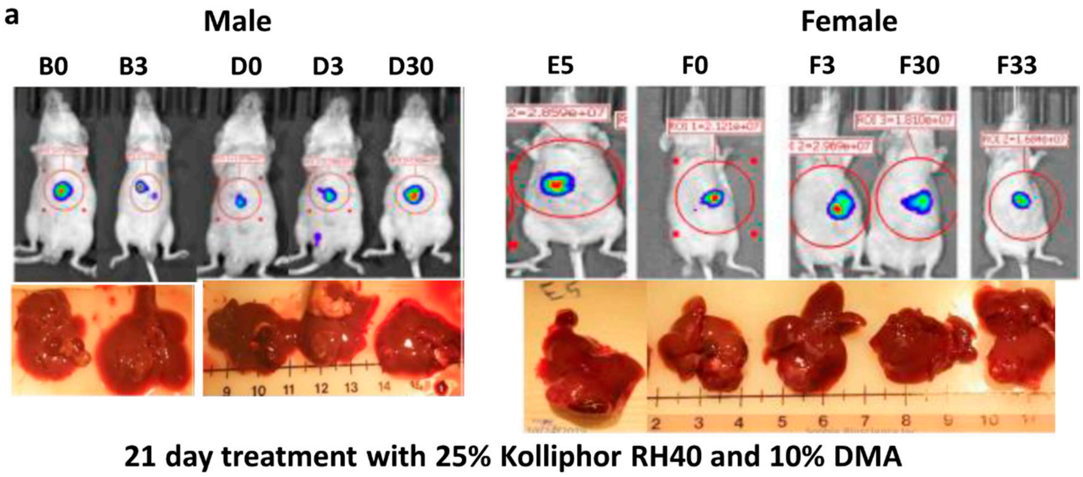

b

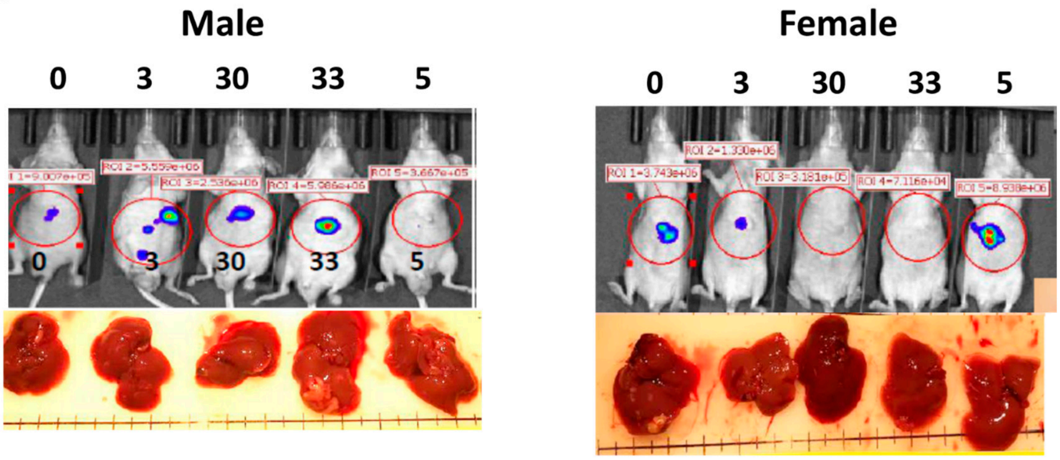

21 day treatment with sorafenib (dosage: 25 mg/kg)

c

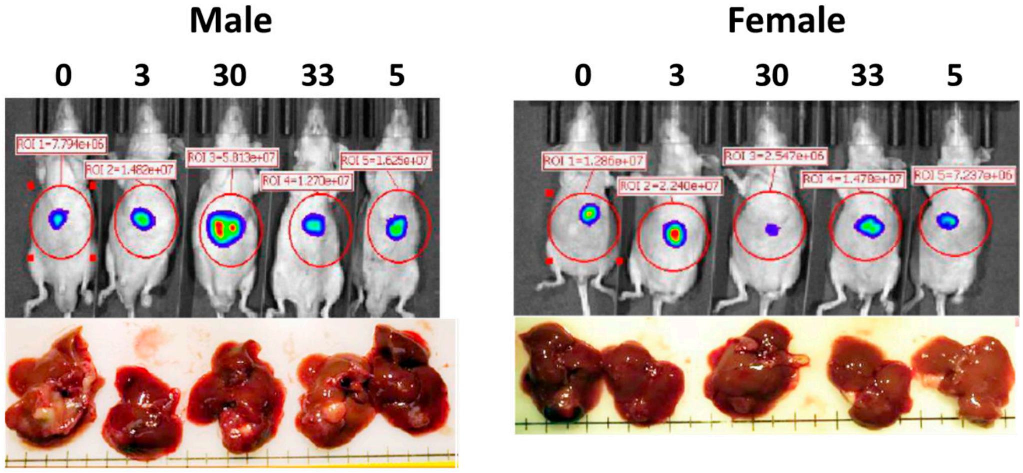

21 day treatment with compound 2 (dosage: 12.5 mg/kg)

d

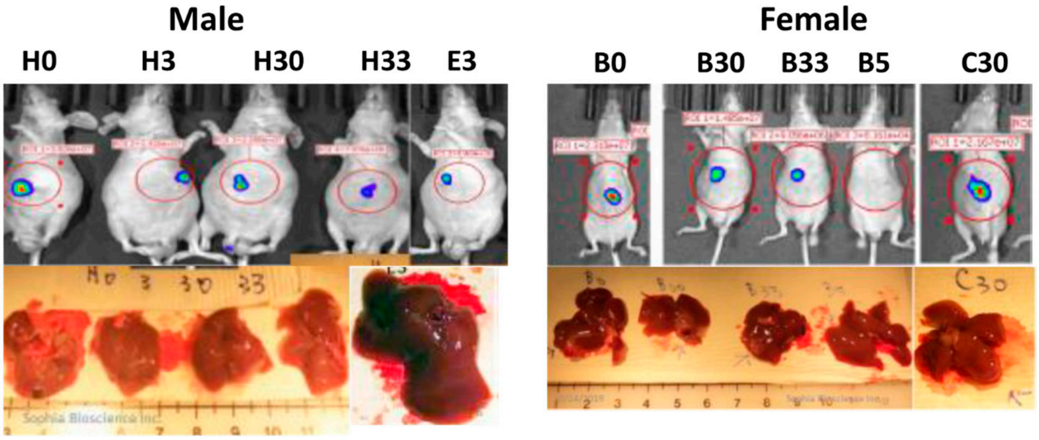

21 day treatment with compound 2 (dosage: 25 mg/kg)

d

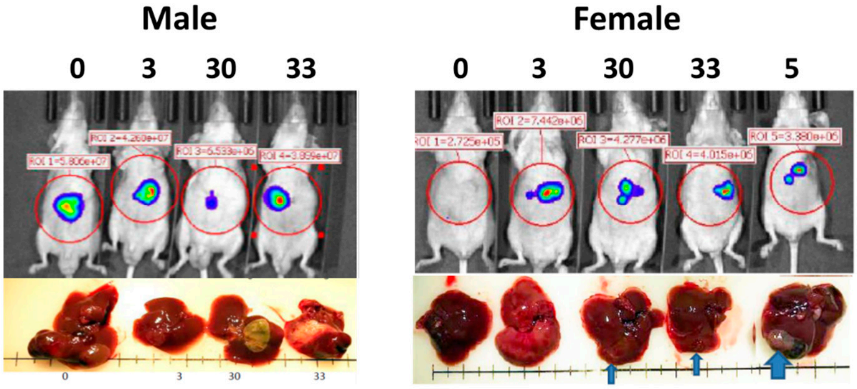

21 day treatment with compound 3a (dosage: 12.5 mg/kg)

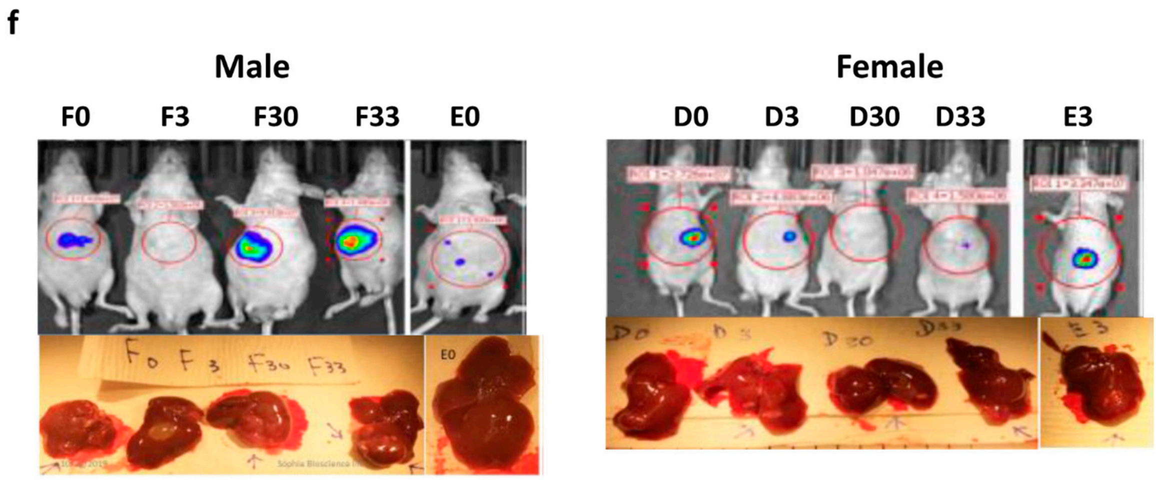

Figure S4: Post-experiment bioluminescent imaging mice (images were captured at day 22nd just before tumors were excised) and pictures of excised mice livers – (a) No treatment (control group); animal cohorts treated with (b) Sorafenib (25 mg/kg), (c) **2** (12.5 mg/kg), (d) **2** (25 mg/kg), (e) **3a** (12.5 mg/kg), (f) **3a** (25 mg/kg).

Compound Synthesis

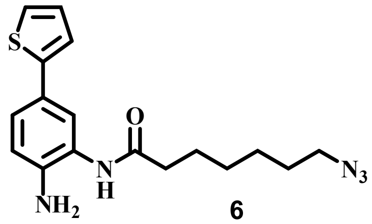

6

N-(2-Amino-5-(cyclopeanta-1,3-dien-1-yl)phenyl-7-azidoheptanamide (**6**). 7-Azidoheptanoic acid **5** (1.02 g, 5.95 mmol) was dissolved in anhydrous DMF (20 mL) and the solution was cooled to 0 ºC. To that cold mixture EDCI·HCl (1.14 g, 5.95 mmol) and HOBT·H_2O (804 mg, 5.95 mmol) were added and the mixture was stirred at 0 ºC for 10 min. A solution of compound **4** (1.73 g, 5.95

mmol) in DMF (10 mL) and *N*,*N′*-diisopropylethylamine (3.1 mL, 17.85 mmol) was added to the cold activated acid solution and the resultant mixture was stirred at room temperature for another 12 h. After that, the reaction was quenched by adding saturated aqueous $NaHCO_3$ (20 mL) solution and then the mixture was transferred into 500 mL separatory funnel and was diluted with 100 mL of ethyl acetate. After shaking couple of times, the aqueous layer was discarded. The organic layer was further washed with 1M HCl (20 mL), water (20 mL), brine (20 mL), and was dried over anhydrous Na_2SO_4, and then filtered. The filtrate was evaporated and the crude was subjected to next reaction without further purification.

The crude was dissolved 20 mL mixture of 1:1 anhydrous dichloromethane (DCM) and TFA and the resultant mixture was stirred at room temperature for 1 h. After that the excess TFA and DCM were removed under vacuum and the crude was re-dissolved in DCM (20 mL) and was washed with saturated aqueous $NaHCO_3$ solution (20 mL), water (20 mL), brine (20 mL) and was dried over anhydrous Na_2SO_4 and then was filtered. The filtrate was evaporated and the crude was purified by column chromatography (silica gel, 7:1 DCM-acetone) to give the title compound **6** (871 mg, 43%) as brown solid. ^1H NMR (400 MHz, $CDCl_3$) δ 7.40 (d, J = 2.1 Hz, 1H), 7.30 (dd, J = 8.3, 2.1 Hz, 1H), 7.22 – 7.15 (m, 2H), 7.13 (dd, J = 3.6, 1.1 Hz, 1H), 7.00 (dd, J = 5.1, 3.6 Hz, 1H), 6.78 (d, J = 8.2 Hz, 1H), 3.26 (t, J = 6.8 Hz, 2H), 2.40 (t, J = 7.5 Hz, 2H), 1.74 (d, J = 7.1 Hz, 2H), 1.60 (d, J = 13.8 Hz, 3H), 1.42 (p, J = 3.6 Hz, 4H). HRMS (ESI) m/z Calcd. for $C_{17}H_{22}N_5OS$ $[M+H^+]$: 344.1540, found for 344.1537.

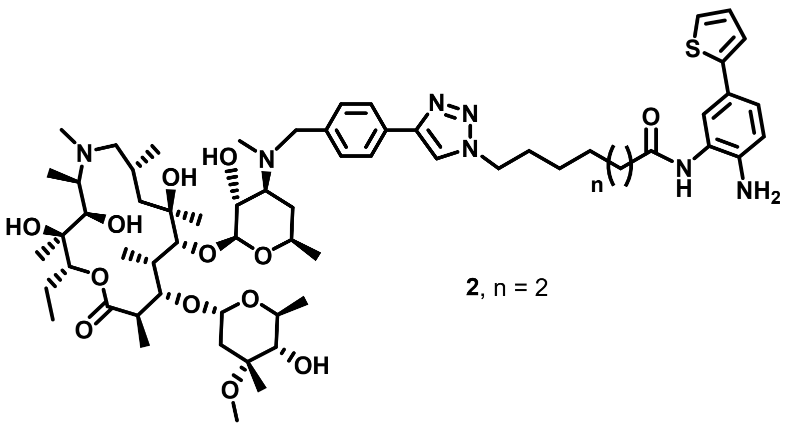

2, n = 2

N-(2-amino-5-(thiophen-2-yl)phenyl)-7-(4-(4-((((2*S*,3*R*,4*S*,6*R*)-2-(((2*R*,3*S*,4*R*,5*R*,8*R*,10*R*,12*S*,13*S*,14*R*)-2-ethyl-3,4,10-trihydroxy-13-(((2*R*,4*R*,5*S*,6*S*)-5-hydroxy-4-methoxy-4,6-dimethyltetrahydro-2*H*-pyran-2-yl)oxy)-3,5,6,8,10,12,14-heptamethyl-15-oxo-1-oxa-6-azacyclopentadecan-11-yl)oxy)-3-hydroxy-6-methyltetrahydro-2*H*-pyran-4-yl)(methyl)amino)methyl)phenyl)-1H-1,2,3-triazol-1-yl)heptanamide (**2**). Azido compound **6** (404 mg, 1.18 mmol) and azithromycin-benzyl alkyne **7** (908 mg, 1.07 mmol) were dissolved in 1:1 DMSO-THF (10 mL) and the solution was degassed by bubbling agron gas. CuI (102 mg, 0.54 mmol) and *N*,*N′*-diisopropylethylamine (0.37 mL, 2.14 mmol) were added to the solution and the resultant mixture was stirred at room temperature for another 12 h. After that, 20 mL solution of 4:1 mixture of saturated NH_4Cl and NH_4OH was added and the mixture was diluted with 100 mL of ethyl acetate. The organic layer was separated and was washed with water (20 mL), brine (20 mL) and was dried over anhydrous Na_2SO_4. The organic layer was filtered and the filtrate was evaporated to dryness. The crude was purified by column chromatography (silica gel, 15% MeOH in DCM) to give the title compound **2** (662 mg, 52%) as light brown solid. ^{1}H NMR (400 MHz, CD_3OD) δ 8.21 (d, J = 3.6 Hz, 1H), 7.65 (d, J = 7.9 Hz, 2H), 7.32 (d, J = 7.9 Hz, 2H), 7.27 (d, J = 2.3 Hz, 1H), 7.18 (dt, J = 8.3, 2.1 Hz, 1H), 7.10 (t, J = 4.2 Hz, 1H), 7.07 – 7.03 (m, 1H), 6.91 – 6.86 (m, 1H), 6.73 (d, J = 8.4 Hz, 1H), 4.88 (s, 1H), 4.37 (dt, J = 12.6, 6.2 Hz, 3H), 4.03 (s, 2H),

3.73 (s, 1H), 3.59 (s, 1H), 3.56 – 3.39 (m, 4H), 3.04 (s, 4H), 2.89 (d, J = 9.7 Hz, 2H), 2.70 (s, 4H), 2.38 – 2.12 (m, 7H), 1.88 (d, J = 8.2 Hz, 3H), 1.72 (d, J = 10.0 Hz, 3H), 1.62 (d, J = 7.8 Hz, 3H), 1.40 (t, J = 12.6 Hz, 6H), 1.28 (s, 5H), 1.16 (d, J = 5.1 Hz, 11H), 1.12 – 0.97 (m, 15H), 0.94 (d, J = 7.3 Hz, 5H), 0.86 (dd, J = 6.8, 2.6 Hz, 4H), 0.77 (qd, J = 9.1, 7.9, 4.4 Hz, 9H). ^{13}C NMR (101 MHz, $CDCl_3$) δ 178.8, 172.2, 147.4, 144.2, 129.7, 129.3, 127.8, 125.7, 124.6, 123.3, 122.9, 121.7, 119.6, 118.2, 102.8, 77.9, 77.7, 74.1, 73.8, 72.7, 70.4, 68.6, 65.6, 57.7, 50.1, 49.3, 45.4, 42.5, 36.8, 36.16, 34.4, 31.9, 29.9, 29.6, 28.2, 27.3, 26.5, 25.9, 25.2, 22.6, 21.9, 21.4, 18.0, 16.4, 14.53, 11.2. HRMS (ESI) *m/z* Calcd. for $C_{63}H_{97}N_7O_{13}S$ $[M+H]^+$: 1192.69378, found 1192.69397.

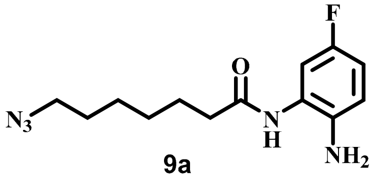

9a

N-(2-amino-5-fluorophenyl)-7-azidoheptanamide (**9a**). 7-Azidoheptanoic acid **5** (1.92 g, 11.21 mmol) and *tert*-butyl (2-amino-4-fluorophenyl)carbamate **8a** (2.79 g, 12.33 mmol) were dissolved in 21 mL of 2:1 (DMF-THF) mixture and to the solution, TBTU (5.40 g, 16.82 mmol) and *N,N'*-diisopropylethylamine (14 mL, 79.59 mmol) were added and the resultant mixture was stirred at room temperature for another 12 h. After that, the reaction was quenched by adding saturated aqueous $NaHCO_3$ (20 mL) solution and then the mixture was transferred into 500 mL separatory funnel and was diluted with 100 mL of ethyl acetate. After shaking couple of times, the aqueous layer was discarded. The organic layer was washed with 1M HCl (20 mL), water (20 mL), brine (20 mL), dried over anhydrous Na_2SO_4, and was filtered. The filtrate was evaporated off under vacuum and the crude residue was subjected to next reaction without further purification.

The crude was dissolved 20 mL mixture of 1:1 anhydrous DCM and TFA and the resultant mixture was stirred at room temperature for 1 h. Subsequently, TFA and DCM were removed under vacuum. The crude was re-dissolved in DCM (20 mL) and washed in succession with saturated aqueous $NaHCO_3$ solution (20 mL), water (20 mL) and brine (20 mL). The organic layer was dried over anhydrous Na_2SO_4, filtered and the filtrate was evaporated off. The crude was purified by column chromatography (silica gel, 7:1 DCM-acetone) to give the title compound **9a** (2.24 g, 71%) as brown solid. ^{1}H NMR (400 MHz, $CDCl_3$) δ 7.46 (s, 1H), 7.22 (dd, J = 11.2, 3.0 Hz, 1H), 6.72 (dd, J = 6.7, 1.6 Hz, 2H), 3.50 (s, 2H), 3.24 (t, J = 6.8 Hz, 3H), 2.34 (dt, J = 13.8, 7.4 Hz, 2H), 1.78 – 1.64 (m, 2H), 1.64 – 1.48 (m, 3H), 1.38 (p, J = 3.7 Hz, 5H). HRMS (ESI) *m/z* Calcd. for $C_{13}H_{19}FN_5O$ $[M+H]^+$: 280.1568, found 280.1564.

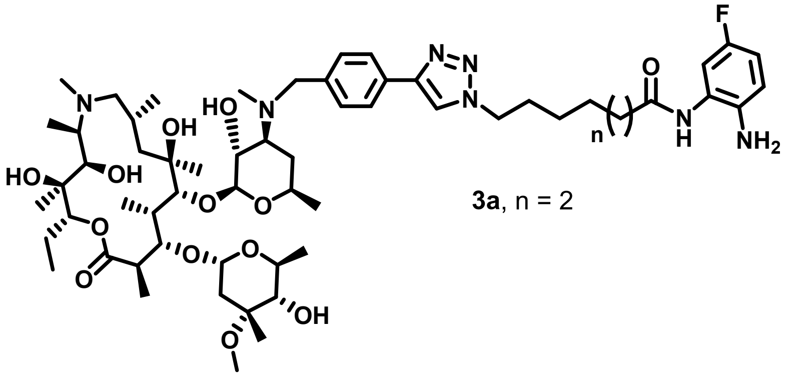

3a, n = 2

N-(2-amino-5-fluorophenyl)-7-(4-(4-((((2*S*,3*R*,4*S*,6*R*)-2-(((2*R*,3*S*,4*R*,5*R*,8*R*,10*R*,12*S*,13*S*,14*R*)-2-ethyl-3,4,10-trihydroxy-13-(((2*R*,4*R*,5*S*,6*S*)-5-hydroxy-4-methoxy-4,6-dimethyltetrahydro-2*H*-pyran-2-yl)oxy)-3,5,6,8,10,12,14-heptamethyl-15-oxo-1-oxa-6-azacyclopentadecan-11-yl)oxy)-3-hydroxy-6-methyltetrahydro-2*H*-pyran-4-yl)(methyl)amino)methyl)phenyl)-1*H*-1,2,3-triazol-1-yl)heptanamide (**3a**). Following the same reaction procedure as described for the synthesis of **2**, reaction between azithromycin-benzyl alkyne **7** (515 mg, 0.61 mmol) and azido compound **9a** (233 mg, 0.68 mmol) in presence of CuI (58 mg, 0.30 mmol) and *N*,*N'*-diisopropylethylamine (0.21

mL, 1.22 mmol) gave title compound **3a** (514 mg, 74%) as light orange solid. ^{1}H NMR (400 MHz, DMSO-d_6) δ 9.09 (s, 1H), 8.55 (s, 1H), 7.76 (d, J = 8.1 Hz, 2H), 7.40 (d, J = 8.1 Hz, 2H), 7.21 (dd, J = 10.7, 2.8 Hz, 1H), 6.77 – 6.63 (m, 2H), 4.85 – 4.68 (m, 4H), 4.39 (t, J = 6.9 Hz, 3H), 4.31 (d, J = 10.9 Hz, 2H), 4.13 (s, 1H), 4.05 (d, J = 7.6 Hz, 3H), 3.76 (d, J = 13.5 Hz, 1H), 3.66 (s, 1H), 3.61 – 3.37 (m, 3H), 3.17 (t, J = 8.4 Hz, 1H), 3.02 (s, 3H), 2.96 – 2.80 (m, 1H), 2.75 – 2.55 (m, 2H), 2.40 – 2.25 (m, 4H), 2.19 (d, J = 8.6 Hz, 8H), 1.88 (t, J = 7.3 Hz, 5H), 1.83 – 1.64 (m, 3H), 1.64 – 1.43 (m, 5H), 1.29 (ddd, J = 31.1, 18.3, 7.9 Hz, 10H), 1.17 (s, 3H), 1.14 (d, J = 6.1 Hz, 4H), 1.11 – 1.06 (m, 5H), 1.04 (d, J = 2.1 Hz, 5H), 1.01 (s, 4H), 0.94 (d, J = 7.3 Hz, 7H), 0.85 (d, J = 6.6 Hz, 4H), 0.78 (t, J = 7.4 Hz, 4H). ^{13}C NMR (101 MHz, $CDCl_3$) δ 171.9, 147.3, 134.9, 129.7, 129.3, 125.6, 119.6, 118.7, 112.1, 110.9, 102.8, 77.9, 77.7, 74.2, 73.9, 72.7, 70.4, 68.6, 65.5, 63.8, 57.7, 50.1, 49.3, 45.3, 42.3, 36.8, 36.4, 36.0, 34.6, 31.5, 30.0, 29.6, 29.0, 28.2, 25.88, 25.1, 22.6, 22.0, 21.3, 20.6, 18.7, 18.0, 16.3, 14.6, 14.1, 11.4, 11.1. HRMS (ESI) *m/z* Calcd. for $C_{59}H_{94}FN_7O_{13}$ $[M+H]^+$: 1128.6966, found 1128.6959.

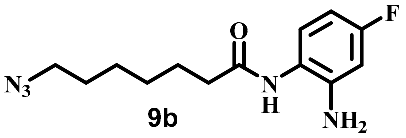

9b

N-(2-amino-4-fluorophenyl)-7-azidoheptanamide (**9b**). Following the same reaction protocol as described for the synthesis of **9a**, 7-azidoheptanoic acid **5** (1.31 g, 7.65) was coupled with aniline compound **8b** (1.93 g, 8.41 mmol) in presence of TBTU (3.76 g, 11.47 mmol) and *N,N′*-diisopropylethylamine (9.40 mL, 54.3 mmol) followed by TFA treatment gave the title compound **9b** (1.56 g, 72%) as light cream solid. ^{1}H NMR (400 MHz, $CDCl_3$) δ 7.02 (dd, J = 8.5, 5.8 Hz, 1H), 6.95 (s, 1H), 6.55 – 6.37 (m, 2H), 3.96 (d, J = 19.9 Hz, 2H), 3.23 (dt, J = 21.2, 6.8 Hz, 2H), 2.45 – 2.30 (m, 2H), 1.73 (d, J = 7.6 Hz, 2H), 1.59 (dd, J = 12.2, 5.9 Hz, 2H), 1.41 (p, J = 3.5 Hz, 4H). HRMS (ESI) *m/z* Calcd. for $C_{13}H_{19}FN_5O$ $[M+H]^+$: 280.1568, found 280.1564.

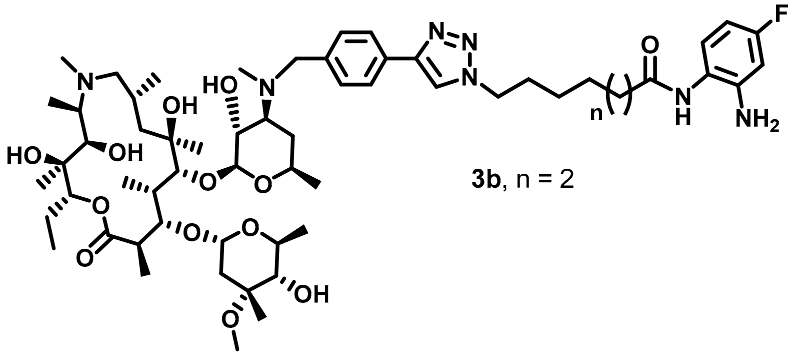

3b, n = 2

N-(2-amino-4-fluorophenyl)-7-(4-(4-(((((2*S*,3*R*,4*S*,6*R*)-2-(((2*R*,3*S*,4*R*,5*R*,8*R*,10*R*,11*R*,12*S*,13*S*,14*R*)-2-ethyl-3,4,10-trihydroxy-13-(((2*R*,4*R*,5*S*,6*S*)-5-hydroxy-4-methoxy-4,6-dimethyltetrahydro-2*H*-pyran-2-yl)oxy)-3,5,6,8,10,12,14-heptamethyl-15-oxo-1-oxa-6-azacyclopentadecan-11-yl)oxy)-3-hydroxy-6-methyltetrahydro-2*H*-pyran-4-yl)(methyl)amino)methyl)phenyl)-1*H*-1,2,3-triazol-1-yl)heptanamide (**3b**). Following the same reaction protocol as described for the synthesis of **2**, azithromycin-benzyl alkyne **7** (505 mg, 0.60) was reacted with azido compound **9b** (228 mg, 0.66) using CuI (57 mg, 0.30) and *N*,*N′*-diisopropylethylamine (0.21 mL, 1.2 mmol) in 4 mL of 1:1 DMSO-THF mixture gave the title compound **3b** (473 mg, 70%) as light cream solid. ^{1}H NMR (400 MHz, DMSO-d_6) δ 8.99 (s, 1H), 8.54 (s, 1H), 7.74 (d, J = 8.0 Hz, 2H), 7.38 (d, J = 8.0 Hz, 2H), 7.06 (dd, J = 8.7, 6.3 Hz, 1H), 6.45 (dd, J = 11.2, 2.9 Hz, 1H), 6.27 (td, J = 8.6, 2.9 Hz, 1H), 5.10 (s, 2H), 4.79 (d, J = 4.7 Hz, 1H), 4.72 (d, J = 9.7 Hz, 1H), 4.37 (t, J = 6.8 Hz, 2H), 4.29 (d, J = 11.5 Hz, 1H), 4.12 (s, 1H), 4.08 – 3.97 (m, 2H), 3.75 (d, J = 13.6 Hz, 1H), 3.64 (s, 1H), 3.58 – 3.36 (m, 2H), 3.16 (t, J = 8.5 Hz, 1H), 3.01 (s, 3H), 2.87 (t, J = 8.4 Hz, 1H), 2.64 (t, J = 6.4 Hz, 2H), 2.55 (s, 2H), 2.34 (d, J = 12.6 Hz, 1H), 2.26 (t, J = 7.4 Hz, 2H), 2.17 (d, J = 7.9 Hz, 6H), 1.95 – 1.77 (m, 4H), 1.65 – 1.38 (m, 5H), 1.24 (d, J = 10.6 Hz, 8H), 1.15 (s, 2H), 1.12 (d, J = 6.0 Hz, 3H), 1.09 – 1.04 (m, 4H), 1.03 (d, J = 2.4 Hz, 3H), 0.99 (s, 3H), 0.93 (dt, J = 6.3, 1.6 Hz, 4H), 0.87 – 0.81 (m, 4H), 0.81 – 0.73 (m, 2H). ^{13}C NMR (101 MHz, $CDCl_3$) δ 178.7, 172.1, 162.8, 160.4, 147.5, 143.1, 138.8, 129.5, 129.3,

127.2, 125.6, 119.9, 119.5, 105.4, 103.8, 102.8, 94.3, 83.5, 78.0, 77.7, 74.0, 73.6, 73.4, 72.7, 70.5, 69.9, 68.6, 65.5, 64.1, 62.4, 57.6, 50.1, 49.3, 45.4, 42.5, 42.1, 36.8, 36.1, 34.5, 29.90, 28.2, 27.7, 26.7, 25.8, 25.2, 22.6, 21.9, 21.3, 18.0, 16.3, 14.5, 11.2, 8.8, 7.0. HRMS (ESI) *m/z* Calcd. for $C_{59}H_{94}FN_7O_{13}$ $[M+H]^+$: 1128.6966, found 1128.6959.

Copy of ^1H, ^{13}C and HPLC spectra of compounds **2**, **3a**, and **3b**

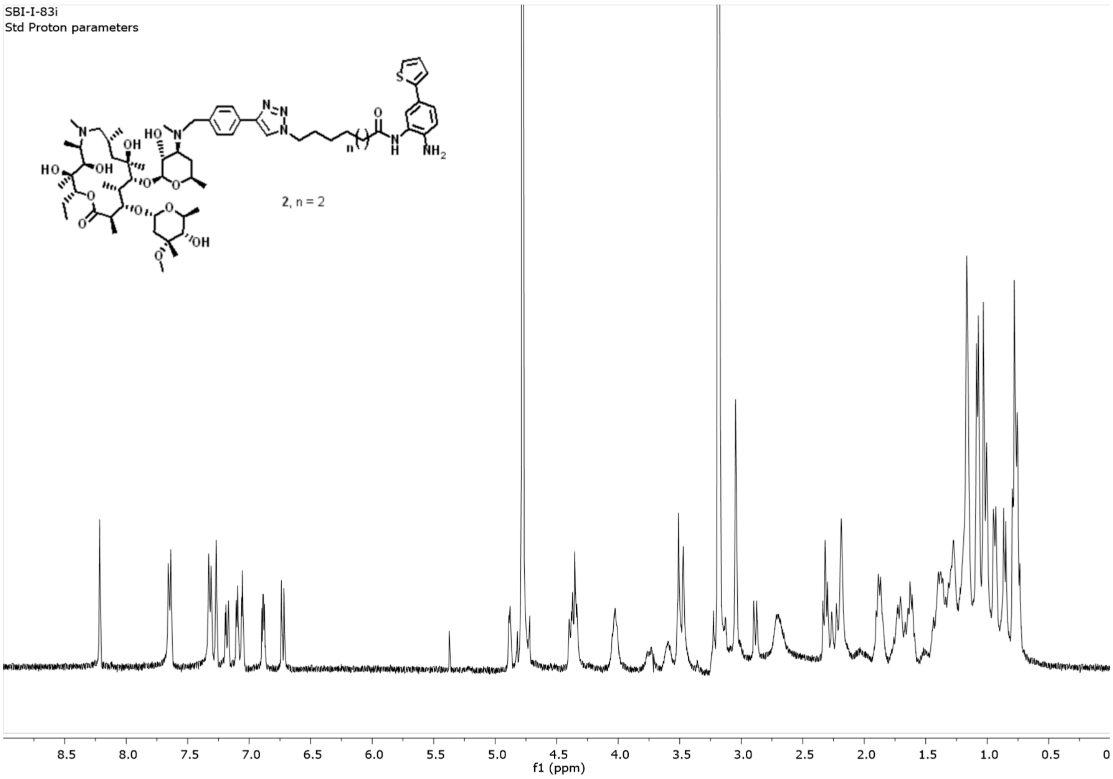

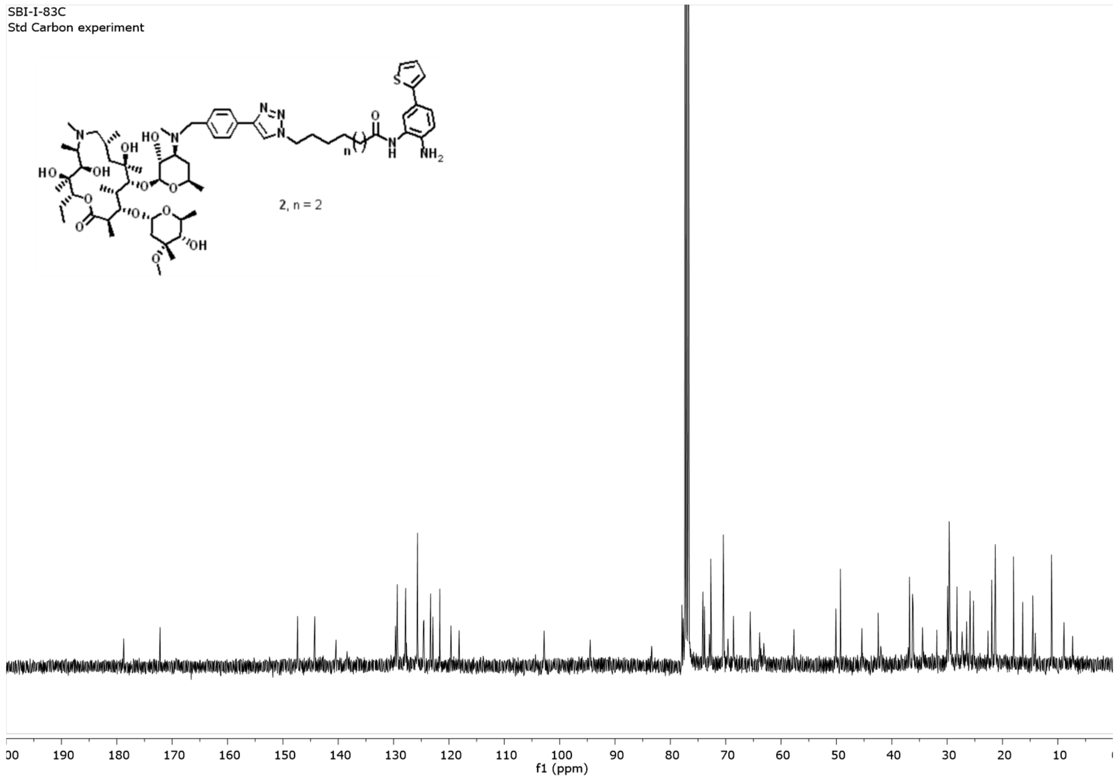
SBI-I-83C
Std Carbon experiment
2, n = 2
f1 (ppm)

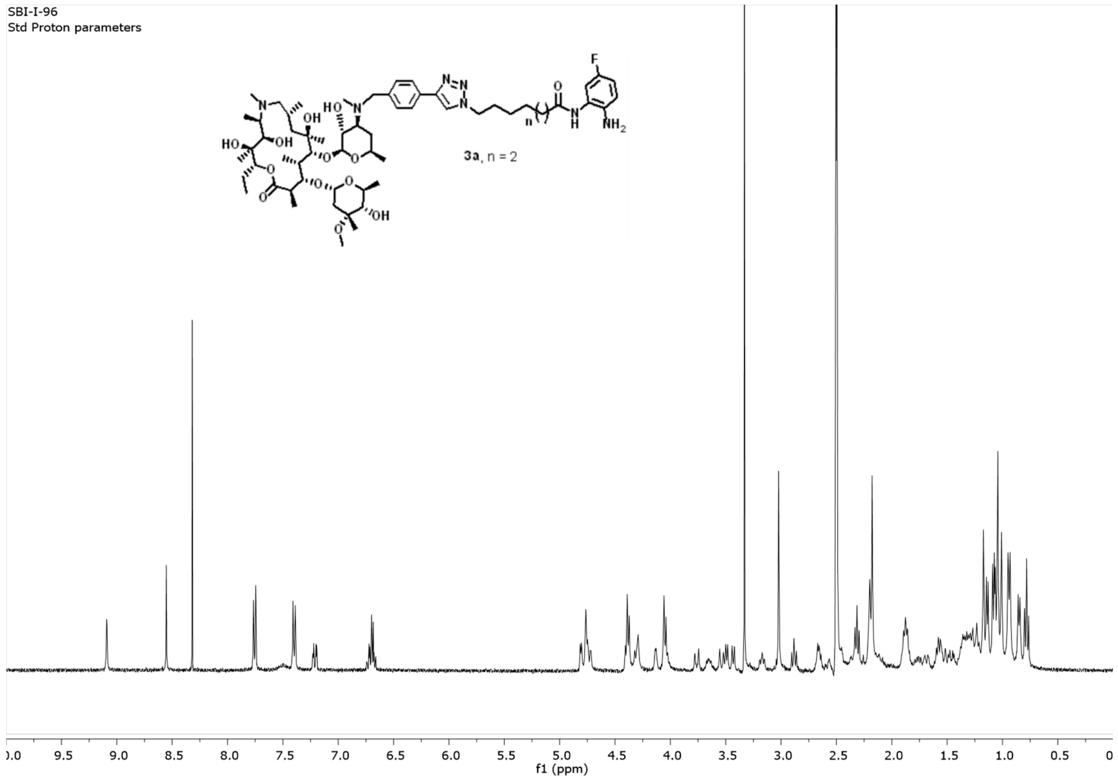
SBI-I-96
Std Proton parameters
3a, n = 2
f1 (ppm)

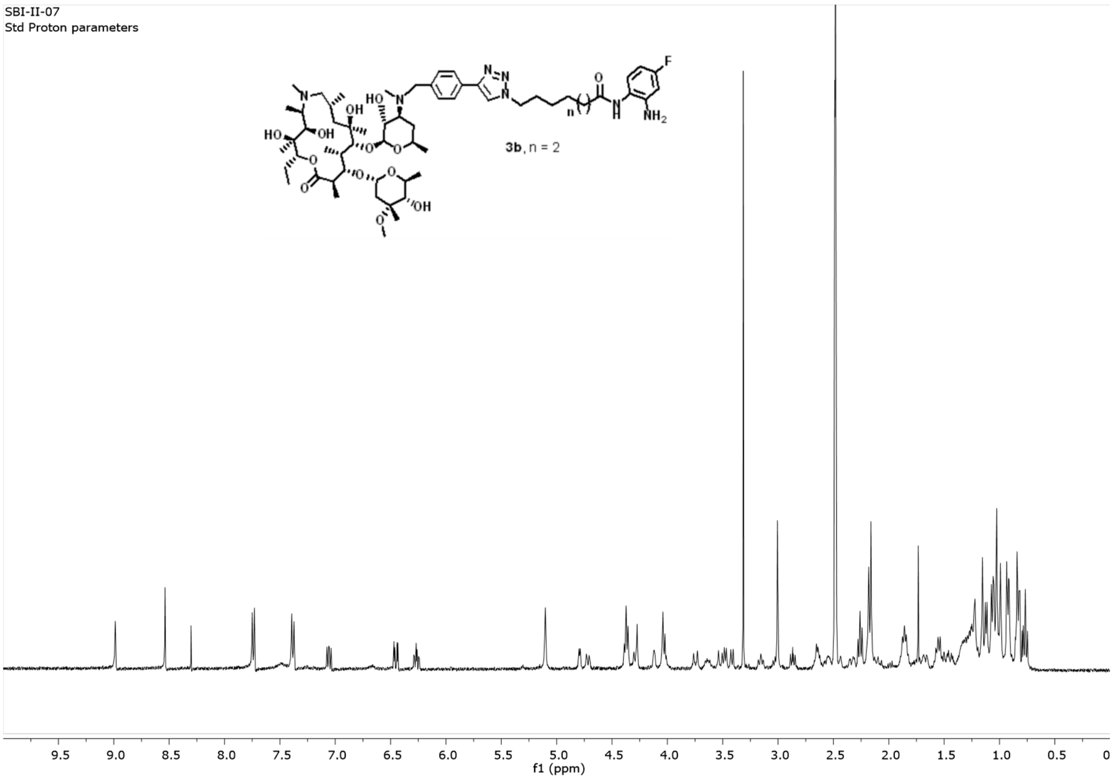
SBI-II-07
Std Proton parameters
3b, n = 2
9.5 9.0 8.5 8.0 7.5 7.0 6.5 6.0 5.5 5.0 4.5 4.0 3.5 3.0 2.5 2.0 1.5 1.0 0.5 0
f1 (ppm)

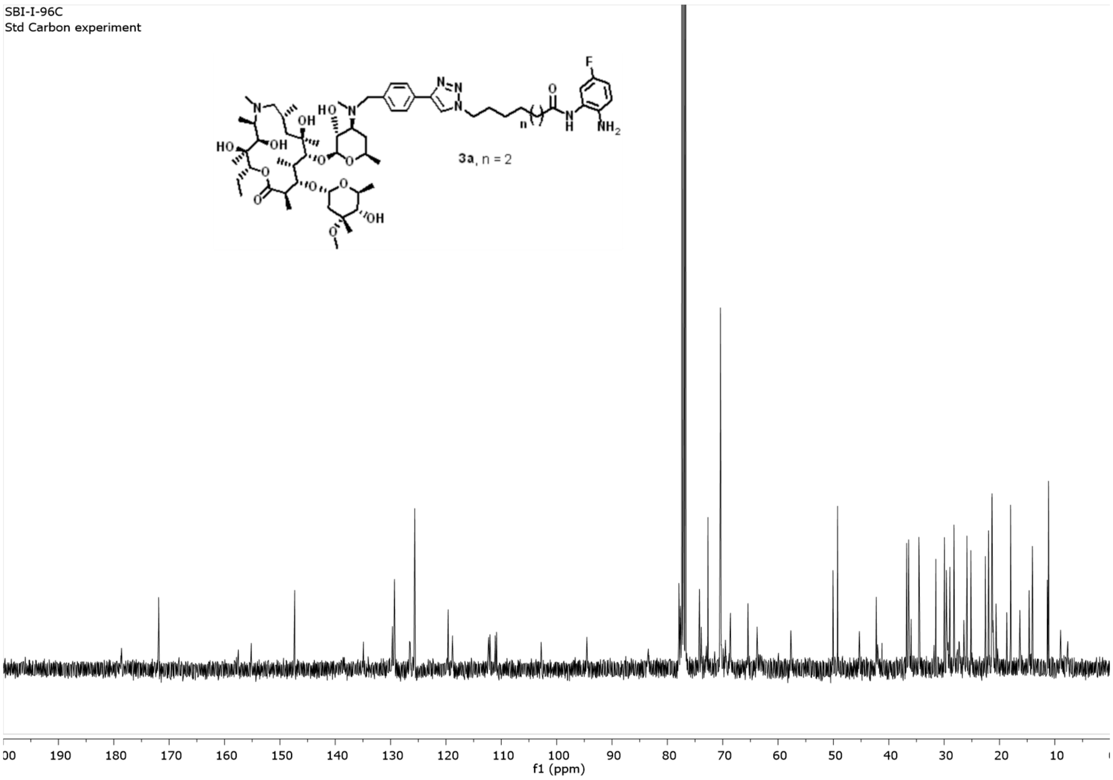
SBI-I-96C
Std Carbon experiment
3a, n = 2
190 180 170 160 150 140 130 120 110 100 90 80 70 60 50 40 30 20 10
f1 (ppm)

SBI-II-07C
Std Carbon experiment

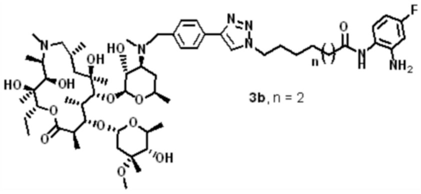

3b, n = 2

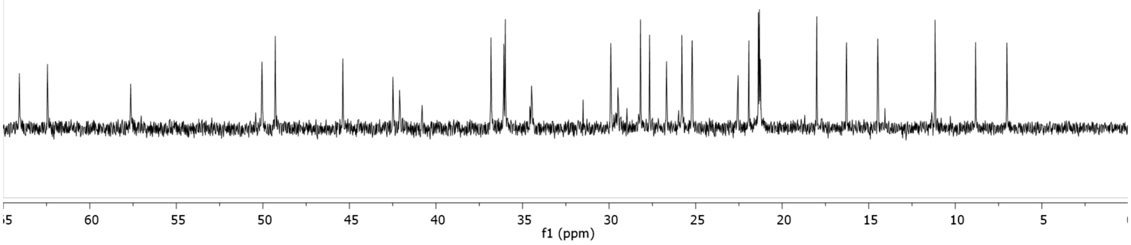

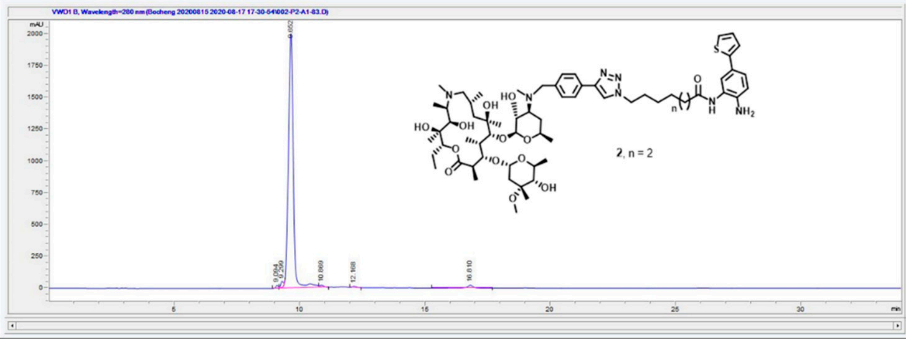

2, n = 2

#	Time	Type	Area	Height	Width	Area%	Symmetry
1	9.094	BV E	159.5	22.8	0.1077	0.566	0.993
2	9.299	VV E	416.4	53.1	0.1214	1.477	1.055
3	9.652	VV R	26872	1998.8	0.1978	95.317	1.195
4	10.869	VB E	208.8	17.3	0.1809	0.741	0.694
5	12.168	BB	96.5	8.8	0.174	0.342	0.689
6	16.81	BB	439.1	23.5	0.2636	1.557	1.257

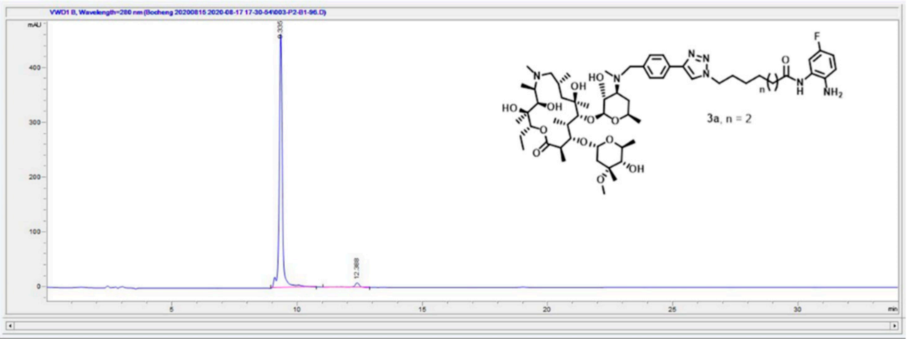

#	Time	Type	Area	Height	Width	Area%	Symmetry
1	9.335	VV R	4056.7	463.4	0.1269	96.875	0.919
2	12.388	VB R	130.8	8.5	0.2189	3.125	1.617

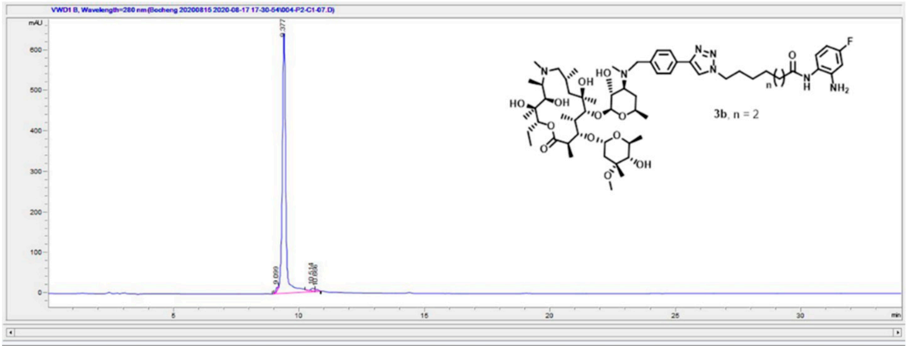

#	Time	Type	Area	Height	Width	Area%	Symmetry
1	9.099	BV E	74.6	13.1	0.0885	1.143	1.477
2	9.377	VV R	6341.2	644.4	0.1468	97.066	0.907
3	10.514	VV E	84.6	7.4	0.1667	1.295	0.907
4	10.666	VB E	32.5	4.5	0.1151	0.497	0.222
